# CD8^+^ T cell/cancer-associated fibroblast ratio stratifies prognostic and predictive responses to immunotherapy across multiple cancer types

**DOI:** 10.3389/fimmu.2022.974265

**Published:** 2022-11-09

**Authors:** Xinlong Zheng, Kan Jiang, Weijin Xiao, Dongqiang Zeng, Wenying Peng, Jing Bai, Xiaohui Chen, Pansong Li, Longfeng Zhang, Xiaobin Zheng, Qian Miao, Haibo Wang, Shiwen Wu, Yiquan Xu, Haipeng Xu, Chao Li, Lifeng Li, Xuan Gao, Suya Zheng, Junhui Li, Deqiang Wang, Zhipeng Zhou, Xuefeng Xia, Shanshan Yang, Yujing Li, Zhaolei Cui, Qiuyu Zhang, Ling Chen, Xiandong Lin, Gen Lin

**Affiliations:** ^1^ Department of Thoracic Oncology, Clinical Oncology School of Fujian Medical University, Fujian Cancer Hospital, Fujian Key Laboratory of Advanced Technology for Cancer Screening and Early Diagnosis, Fuzhou, China; ^2^ Department of Pathology, College of Clinical Medicine for Oncology, Fujian Medical University, Fuzhou, China; ^3^ Department of Oncology, Southern Medical University, Guangzhou, China; ^4^ The Second Department of Oncology, Yunnan Cancer Hospital, The Third Affiliated Hospital of Kunming Medical University, Yunnan Cancer Center, Kunming, China; ^5^ R&D Department, Geneplus-Beijing Institute, Beijing, China; ^6^ Department of Thoracic Surgery, Clinical Oncology School of Fujian Medical University, Fujian Cancer Hospital, Fuzhou, China; ^7^ Chinese People’s Liberation Army 92403 Unit Support Department, Navy Fujian Base Hospital, Fuzhou, China; ^8^ Department of Medical Genetics and Genomics, National Protein Science Center, Beijing, China; ^9^ Department of Medical Oncology, Cancer Therapy Center, Affiliated Hospital of Jiangsu University, Zhenjiang, China; ^10^ Laboratory of Biochemistry and Molecular Biology Research, Department of Clinical Laboratory, Fujian Cancer Hospital, Fujian Medical University Cancer Hospital, Fuzhou, China; ^11^ Institute of Immunotherapy, Fujian Medical University, Fuzhou, Fujian, China; ^12^ Laboratory of Radiation Oncology and Radiobiology, Clinical Oncology School of Fujian Medical University, Fujian Cancer Hospital, Fuzhou, China

**Keywords:** CD8+ T cell, cancer-associated fibroblast, prognostic biomarker, predictive biomarker, immunotherapy

## Abstract

**Background:**

Cancer-associated fibroblasts (CAFs) within the tumor microenvironment (TME) are critical for immune suppression by restricting immune cell infiltration in the tumor stromal zones from penetrating tumor islands and changing their function status, particularly for CD8^+^ T cells. However, assessing and quantifying the impact of CAFs on immune cells and investigating how this impact is related to clinical outcomes, especially the efficacy of immunotherapy, remain unclear.

**Materials and methods:**

The TME was characterized using immunohistochemical (IHC) analysis using a large-scale sample size of gene expression profiles. The CD8^+^ T cell/CAF ratio (CFR) association with survival was investigated in The Cancer Genome Atlas (TCGA) and Gene Expression Omnibus (GEO) lung cancer cohorts. The correlation between CFR and immunotherapeutic efficacy was computed in five independent cohorts. The correlation between CFR and objective response rates (ORRs) following pembrolizumab monotherapy was investigated in 20 solid tumor types. To facilitate clinical translation, the IHC-detected CD8/α-SMA ratio was applied as an immunotherapeutic predictive biomarker in a real-world lung cancer cohort.

**Results:**

Compared with normal tissue, CAFs were enriched in cancer tissue, and the amount of CAFs was overwhelmingly higher than that in other immune cells. CAFs are positively correlated with the extent of immune infiltration. A higher CFR was strongly associated with improved survival in lung cancer, melanoma, and urothelial cancer immunotherapy cohorts. Within most cohorts, there was no clear evidence for an association between CFR and programmed death-ligand 1 (PD-L1) or tumor mutational burden (TMB). Compared with TMB and PD-L1, a higher correlation coefficient was observed between CFR and the ORR following pembrolizumab monotherapy in 20 solid tumor types (Spearman’s r = 0.69 *vs.* 0.44 and 0.21). In a real-world cohort, patients with a high CFR detected by IHC benefited considerably from immunotherapy as compared with those with a low CFR (hazard ratio, 0.37; 95% confidence interval, 0.19–0.75; *p* < 0.001).

**Conclusions:**

CFR is a newly found and simple parameter that can be used for identifying patients unlikely to benefit from immunotherapy. Future studies are needed to confirm this finding.

## Introduction

The tumor microenvironment (TME) comprises a wide array of immune and stromal cells, cytokines, chronic inflammation, and immunosuppression, and pro-angiogenic intratumoral atmosphere is highly related to clinical outcomes and treatment efficacy (1, 2).

As the most abundant stromal cells in the TME, cancer-associated fibroblasts (CAFs) promote tumor progression *via* multiple pathways and have a core role in dampening the immune response to cancer (3). Prior studies have focused on a novel mechanism of CAF-mediated T-cell depletion and dysfunction within tumors (4, 5). CD8^+^ T-cell infiltration and cytotoxicity are the most salient determinants of anti-tumor immunity (6). CAFs could abrogate CD8^+^ T-cell function *via* the perpetually secreted extracellular matrix, which produces a dense web of collagen, restricting T-cell trafficking to the tumor, reducing T-cell infiltration into tumor islands, and suppressing the cytotoxic function of T cells (7, 8). Notably, T cells react to CAF signaling and establish bidirectional crosstalk. When T cells are trapped in the stroma, they upregulate inhibitory molecules on the CAF surface, potentially limiting their residual function (9, 10). CAFs are a significant impediment to multiple immune populations across several cancer types, especially for effective cytotoxic T-cell immunity.

Although the complex crosstalk between CAFs and immune cells is well-known, assessing the interaction between CAFs and immune cells and how this interaction is related to clinical outcomes, especially the immunotherapeutic efficacy, remains unclear. Recent advances in genomic sequencing and bioinformatics have enabled high-throughput analysis and interpretation of complex disease-related datasets. These are ideal approaches to quantify tumor-infiltrating immune cells and other cells in the TME of various cancers (11–13). Based on gene expression profiles, a recent study employed computational algorithms to predict immune-checkpoint blockade responses by estimating TME infiltration patterns of 1,524 tumors among gastric cancer patients (14). This approach was adopted to explore the clinical utility of TME infiltration and has brought about promising developments in treating other types of cancer (15, 16). The clinical practicality of these biomarkers based on mathematical models is limited by their multiparametric nature and excessive complexity. Nonetheless, a simple and readily available biomarker has gained widespread clinical applicability in a recent study on surgically resected colon cancer, wherein the interaction between FAP+ fibroblasts and CD8^+^ T cells significantly facilitated the cancer diagnosis (17). The relationship between the TME and immunotherapy remains unexplored.

Driven by the underlying mechanism and pressing clinical needs, the TME in this study was characterized by immunohistochemical (IHC) analysis of clinical samples and a publicly available large sample size of gene expression profiles. Considering the potential negative effects that CAFs cause on immune cells, we sought to better assess the immunomodulatory processes of the TME. First, we generated a novel parameter called CD8^+^ T cell/CAF ratio (CFR) to evaluate the interaction between CD8^+^ T cells and CAFs. Next, the significance of biomarker CFR in predicting prognosis and immunotherapeutic efficacy in the pan-cancer milieu was explored.

## Materials and methods

### Study design and patient datasets

**Figure 1:**
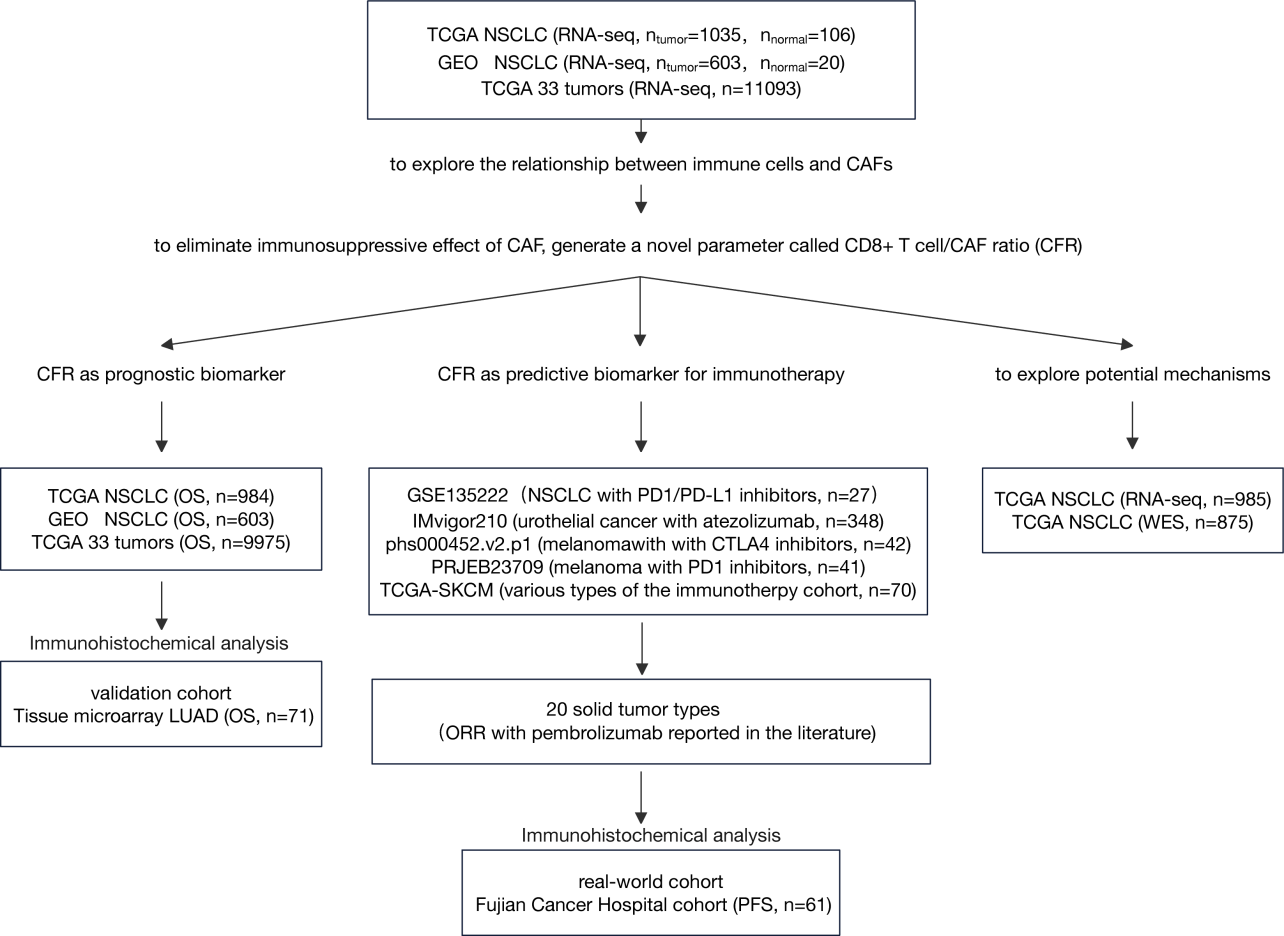
Flow diagram of the study. LUAD, lung adenocarcinoma; NSCLC, non-small cell lung cancer; IHC, immunohistochemistry; CAF, cancer-associated fibroblast; ORR, objective response rate; OS, overall survival.

We analyzed the expression profiles of CAFs and immune cells from multiple independent cohorts. Subsequently, we analyzed the prognostic and predictive roles of CD8^+^ T cell/CFR across these cohorts, as illustrated in **Figure 1**.

Three cohorts, The Cancer Genome Atlas (TCGA) non-small cell lung cancer (NSCLC) cohort, Gene Expression Omnibus (GEO) GLP570 platform NSCLC cohort, and TCGA 33 tumors cohort, were used to explore the relationship between immune cells and CAFs and investigate CFR as an independent prognosis factor. RNA sequencing (RNA-seq) data by fragments per kilobase of exon model per million reads mapped (FPKM) normalization were downloaded from TCGA platform. FPKM values were converted to transcripts per million values (18). Normalized gene expression data for pan-cancer comparisons were downloaded from the UCSC Xena platform (https://xenabrowser.net/). All GEO data (GSE31210, GSE37745, and GSE50081) were downloaded as original counts and were standardized using the Robust Multi-array Average algorithm. The ComBat was used for batch correction (19). The clinical information of the three cohorts is summarized in **Supplementary Table 1**. As an independent non-public validation cohort, tissue microarray (cat. no. HlugA180Su04) comprised 71 pairs of lung adenocarcinoma tissues and matched normal adjacent tissues with the clinicopathological data summarized in **Supplementary Table 2**, which was provided by Shanghai Outdo Biotech Co. Ltd. (Outdo Biotech). These cohorts had sufficient follow-up data for overall survival (OS). TCGA cancer-type abbreviations can be found at https://gdc.cancer.gov/resources-tcga-users/tcga-code-tables/tcga-study-abbreviations.

To determine the association between CFR and the outcome of patients who received immunotherapy, we included five public immune checkpoint inhibitors (ICIs) datasets, namely, IMvigor210 (urothelial cancer treated with atezolizumab), PRJEB23709 (melanoma treated with programmed cell death protein 1 (PD-1) inhibitors), phs000452.v2.p1 (melanoma treated with CTLA4 inhibitors), GSE135222 (lung cancer treated with PD-1/programmed death-ligand 1 (PD-L1) inhibitors), and TCGA-SKCM (various types of the immunity treatment cohort, including CTLA4 inhibitors). The clinical information of these cohorts is summarized in **Supplementary Table 3**. We evaluated the relationship between CFR and available objective response data for pembrolizumab therapy in major solid tumor types of TCGA pan-cancer cohort. Details on the methods are provided in the **Supplementary Material**. To translate the previous *in silico* findings into clinical samples, we selected 61 patients with advanced NSCLC treated with anti-PD-1/PD-L1 therapy as a standard practice or in a clinical trial; this took place at Fujian Cancer Hospital from November 2016 to August 2021. The clinical information of the Fujian Cancer Hospital cohort (FCHC) is summarized in **Supplementary Table 4**. Ethical approval was obtained through the Ethics Committee of Fujian Cancer Hospital. These cohorts for predictive analysis possessed at least one type of survival data [progression-free survival (PFS)] for immunotherapy.

### Genetic signatures

The marker genes for the immune cell types were obtained from Bindea et al. (20) Twenty four immune cell types were involved in innate immunity (natural killer [NK] cells, NK CD56^dim^ cells, NK CD56^bright^ cells, dendritic cells [DCs], plasmacytoid DCs [pDCs], immature DCs [iDCs], activated DCs [aDCs], neutrophils, mast cells, eosinophils, and macrophages) and adaptive immunity (B cells, T cells, CD8 T cells, T helper [Th], Th1, Th2, Th17, T gamma delta [Tgd], T central memory [Tcm], T effector memory [Tem], T follicular helper [Tfh], Tregs, and cytotoxic cells). CD8+ T cells (DNAJB1, DNAJB1, ZFP36L2, ZFP36L2, VAMP2, PPP1R2, TBCC, LEPROTL1, CAMLG, KLF9, GADD45A, CD8A, ZNF91, PF4, THUMPD1, TSC22D3, SLC16A7, GZMM, ZEB1, RBM3, APBA2, C4orf15, SF1, FLT3LG, C19orf6, ZNF609, SFRS7, PRF1, TMC6, MYST3, AES, ZNF22, ABT1, CDKN2AIP, ARHGAP8, LIME1, PRR5, and C12orf47) and other immune cell gene signatures were obtained from Bindea et al. (20) The CAF gene signatures (COL1A1, COL3A1, COL6A1, COL6A2, DCN, GREM1, PAMR1, and TAGLN) were obtained from the Microenvironment Cell Populations Counter (Ebecht on GitHub [https://github.com/ebecht]) (12). In this study, all gene signatures are provided in **Supplementary Table 5**.

The infiltration levels and immune scores of the immune cell types were introduced to evaluate the composition of the tumor immune infiltration. Single-sample gene set enrichment analysis (ssGSEA) was implemented using the R package GSVA to quantify the relative infiltration of immune cells and CAFs (20, 21). Generally, ssGSEA is a rank-based method that computes an overexpression measure for a gene list of interest relative to all other genes within the genome. Immune scores were calculated using the ESTIMATE method to characterize the degree of immune infiltration (13).

### CD8^+^ T cell/cancer-associated fibroblast ratio and immune cell/cancer-associated fibroblast ratio

To calculate the potential negative effect of CAFs on CD8^+^ T cells, a novel parameter referred to as CD8^+^ T cell/CFR was employed. Since many other subtypes of immune cells exist apart from CD8^+^ T cells, the term immune cell/CFR (ICFR) was defined for convenience. This study applied CFR to represent the numerical difference between the ssGSEA scores of CD8^+^ T cells and CAFs. The calculation method was set up according to the principles described in a prior work (22). The same calculation was performed to quantify ICFRs.

In clinical samples, CFR was defined as the area ratio of CD8^+^ T cells to CAFs determined by immunohistochemical staining of CD8 and α-SMA. In the lung adenocarcinoma (LUAD) tissue microarray cohort, regions of the equal area were selected for quantification, which could ensure that the same amount of tissue was considered in each slide. In FCHC, one to three regions were collected, excluding blood vessels and necrotic areas, and the total area was calculated as the final result; the percentage of CD8^+^ T-cell area or CAF area was normalized against the total area for each sample. The quantification results are summarized in **Supplementary Table 6**. The immunohistochemically stained slides were briefly scanned on a Motic Digital Slide Scanner and analyzed *via* Motic VM 3.0. Under ×100 magnification, we selected tumor bed areas (including the tumor epithelium and intratumoral stroma), except for necrosis and blood vessels, and then we calculated the selected areas using ImageJ software (ImageJ; National Institutes of Health; http://rsbweb.nih.gov/ij).

### Immunohistochemistry

Formalin-fixed and paraffin-embedded specimens were sectioned at a thickness of 3 μm, dewaxed, rehydrated in graded ethanol, and immunohistochemically stained for CD8 (1:100, clone SP57, Dako M7103, Dako, Glostrup, Denmark) and α-SMA (1:100, clone 1A4, Dako M0755). Antigen retrieval was performed with Tris-HCl (pH 9) for 30 min at 95°C. Antibody testing and staining protocols were established, and staining was performed by an automated Leica BOND RXsystem (Leica BOND RX, Leica Biosystems, Wetzlar, Germany) with the Bond Polymer Refine Detection Kit (with DAB as chromogen) and Bond Polymer Refine Red Detection Kit for the double staining (Leica Biosystems). CAFs were not only defined by IHC but were further confirmed by microscopy based on morphological criteria.

PD-L1 expression was verified by IHC using the Dako PD-L1 IHC 22C3 pharmDx assay (Agilent Technologies, Santa Clara, CA, USA). The tumor proportion score (TPS) of PD-L1 was computed as the percentage of at least 100 viable tumor cells with complete or partial membrane staining (23). The pathologists of the commercial vendor provided TPS interpretation.

### Gene set enrichment analysis

For gene set enrichment analysis (GSEA) of RNA-seq data, the false discovery rate (FDR) and enrichment score (ES) were computed using GSEA 4.2.0 Java software (24). The analysis was executed using the curated “Hallmark” signature collection from the Molecular Signatures Database (MSigDB) (25).

### Statistical analyses

The normality of the variables was assessed with the Shapiro–Wilk normality test. The statistical significance of the normally and non-normally distributed variables was estimated using the unpaired Student’s *t*-test and the Mann–Whitney *U* test, respectively, to compare the two groups. The Kruskal–Wallis test and the one-way analysis of variance (ANOVA) were used as non-parametric and parametric methods to compare multiple groups. The correlation coefficient was computed using Spearman’s test and the distance correlation analyses. Categorical variables were compared by chi-square analysis, continuous correction chi-square test or Fisher’s exact test, and continuous variables by Wilcoxon test. In each independent cohort, the optimal cut-points for CFR were determined by maximally selected rank statistics (26) using the *surv cut-point* function of R package *survminer* for best CFR^high^ and CFR^low^ selection. Under the optimal cutoff point, the R package *forestplot* was employed to demonstrate the results of the group analysis of CFR in NSCLC and TCGA pan-cancer datasets. The Kaplan–Meier method was used to generate survival curves for the groups from each dataset, and the log-rank Mantel–Cox test was used to determine the statistical significance of the differences. The univariate hazard ratio (HR) was calculated using a univariate Cox proportional hazards regression model. Fitting multivariate models were performed using logistic regression. The areas under the receiver operating characteristic (ROC) curves (AUCs) were estimated non-parametrically. With the use of the *survminer* software package, the multiple Cox regression model was applied to identify the independent prognostic factors. R software was employed for the statistical analyses. A *p*-value <0.05 was considered statistically significant.

## Results

### Moderately positive correlation between cancer-associated fibroblasts and immune cell infiltration

**Figure 2:**
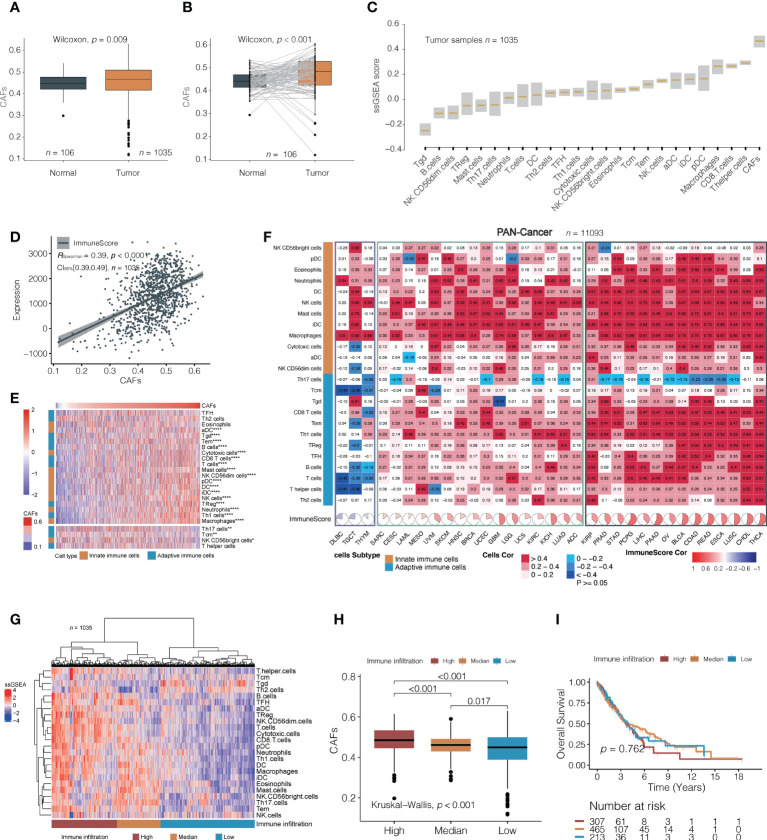
A correlation between cancer-associated fibroblasts (CAFs) and immune cells. **(A, B)** Analysis of TCGA NSCLC cohorts found that cancer-associated fibroblasts (CAFs) were significantly higher in tumor tissues than in normal (median value; A: 0. 460 *vs.* 0. 441; B: 0.484 *vs.* 0.441). **(C)** The relative amounts of CAFs and different immune cell types in TCGA NSCLC cohort. The lower and upper bounds of the box in a boxplot indicate the first and third quartiles, respectively. Cell types are ordered by their median ssGSEA scores. **(D)** A correlation between CAFs and immune score in TCGA NSCLC cohorts. **(E)** The non-clustering heat map is arranged according to the correlation coefficient between CAF and 24 types of immune cells in TCGA NSCLC cohorts; Spearman’s test. The colors from red to blue denote their correlation coefficients of the immune score and CAFs from high to low. The top part indicates immune cells positively correlated with the content of fibroblasts, and the bottom part shows negative correlations. *, *p* < 0.05; **, *p* < 0.01; ****, *p* < 0.001. **(F)** The correlation between CAFs and 24 immune cells in 33 independent pan-cancer cohorts, clustering tumor types from left to right according to the correlation coefficient between CAFs and immune score. The black box indicates a positive correlation between CAFs with most immune cells, the yellow box indicates a positive correlation between CAFs with most innate immune cells, and the blue box indicates a negative correlation between CAFs and distinct immune cells. **(G)** CAF difference analysis between high, medium, and low tumors with immune infiltration in TCGA NSCLC cohorts. The clusters were generated using unsupervised K-means clustering. **(H, I)** Differences in CAFs (median value; high, 0.485; median, 0.461; low, 0.449) and overall survival (median value; high, 3.66, median, 3.89; low, 3.98) among the three immune infiltration levels were analyzed in TCGA NSCLC cohorts.

The flowchart of this study is shown in **Figure 1**. To systematically investigate TME cell infiltration, ssGSEA was performed in TCGA (discovery set) and GEO (validation set) NSCLC cohorts. CAFs were specifically enriched in total NSCLC tumor samples compared to normal samples (Wilcoxon test, *p* = 0.009; **Figure 2A** and **Supplementary Figure 1A**). Additionally, pairing analysis of the normal and tumor samples derived from the same patient confirmed the differential enrichment of CAFs in normal and tumor tissues (Wilcoxon matched pairs test, *p* < 0.001; **Figure 2B**). Notably, the abundance of CAFs is overwhelmingly higher in tumor samples than in other immune cells (**Figure 2C** and **Supplementary Figure 1B**).

We first explored CAFs and their relationships with comprehensive immune status and observed a positive correlation between CAFs and the ImmuneScore based on the ESTIMATE algorithm (Spearman’s correlation test; TCGA: r = 0.44, 95% confidence interval [CI], 0.35–0.49, *p* < 0.001; GEO: r = 0.39, 95% CI, 0.32–0.46, *p* < 0.001; **Figure 2D**, **Supplementary Figure 1C**). We then investigated the relationship between CAFs and 24 distinct immune cells. Surprisingly, CAFs were positively correlated with nearly all immune cells, except for Th17 (**Figure 2E**; **Supplementary Figure 1D** and **Supplementary Table 7**). Thirty-three independent TCGA cancers were subjected to pan-cancer analysis to determine whether the correlation between immune cell infiltration and CAFs was consistent across pan-cancers. Most tumor types presented a positive correlation, except for diffuse large B-cell lymphoma, testicular germ cell tumors, and thymoma, where multiple immune cells exhibited no significant association with CAFs (**Figure 2F**). In pan-cancer cohorts, the correlation between CAFs and immune cell infiltration can be roughly categorized into two subgroups: one with both adaptive and innate immune cells positively associated with CAFs and the other with a predominance of innate immune cells associated with CAFs. The Th17 were either of no significant correlation or negatively correlated with CAFs in the majority of pan-cancer cohorts, which is consistent with the results in TCGA and GEO cohorts. These observations may suggest the existence of interactions between CAFs and distinct immune cells in regulating the TME. Overall, the moderately positive correlation between them is a common feature of cancers.

Next, unsupervised clustering partitioned the tumor samples into high, medium, and low immune infiltration subgroups (**Figure 2G** and **Supplementary Figure 1E**). It was observed that CAF content decreased from a high to low immunity (Kruskal–Wallis test, overall *p* < 0.001, **Figure 2H**; **Supplementary Figure 1F**). Although high-level immune cell infiltration exhibited higher cytolytic activity (CYT) (27) and interferon-gamma (INF-γ) (28), the probabilities of OS were not statistically significantly different among the three groups (log-rank test; TCGA: overall *p* = 0.762; GEO: overall *p* = 0.147) (**Figure 2I**; **Supplementary Figures 1G, H**).

### CD8+ T cell/Cancer-associated fibroblast ratio as an independent favorable prognostic factor for pan-cancer

**Figure 3:**
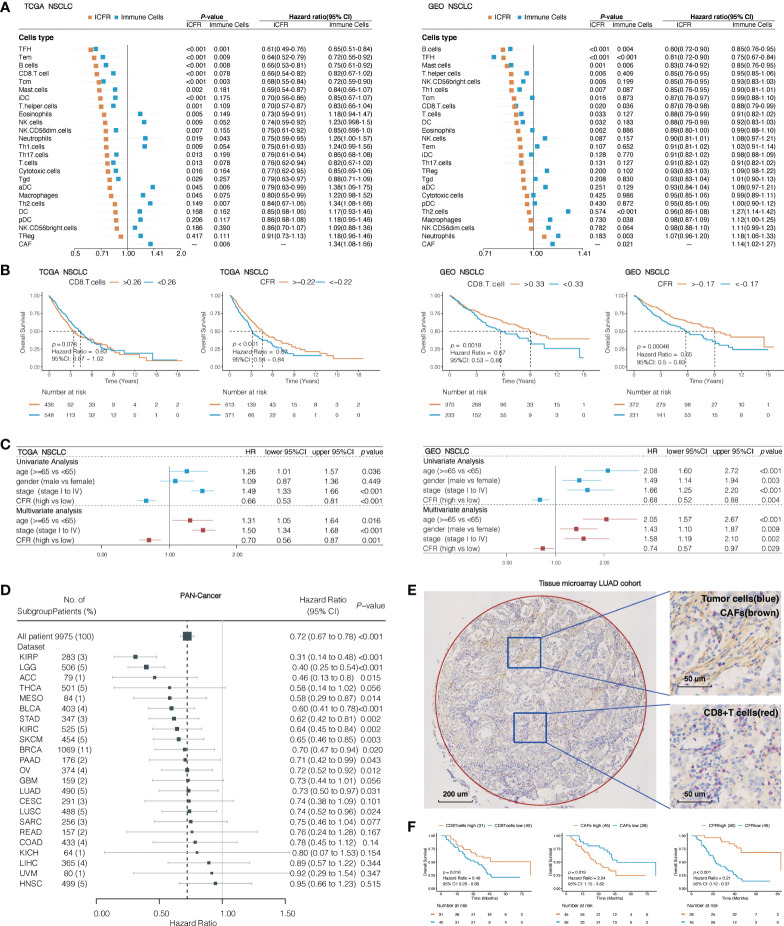
Association between immune cells/CAF ratio (ICFRs) and overall survival in lung cancer and pan-cancer cohort. **(A)** Prognostic value of 24 types of immune cells, CAFs, and ICFRs in non-small cell lung cancer from TCGA and GEO datasets. **(B)** Kaplan–Meier OS curves of CD8 T cells and CD8^+^ T cell/CAF ratio (CFR) in TCGA and GEO lung cancer cohort. **(C)** Univariate and multivariate analyses of the association between CFR and prognosis. **(D)** Association between CFR and overall survival in pan-cancer. Cancer types with abnormal or excessive confidence intervals were excluded, and only 23 cancer types were displayed. **(E)** Representative images indicated double staining for α-SMA (brown) and CD8 (red). The circle represents the sweep region. **(F)** Kaplan–Meier survival curves showing the effect of α-SMA, CD8, and CD8/α-SMA.

To understand the impact of CAFs, we independently evaluated the effects of immune cells and ICFRs on prognosis. First, single-parameter distinct immune cells or CAFs associated with prognosis were evaluated in TCGA and GEO NSCLC cohorts. In the univariate analysis, only CAFs, Th2 cells, and neutrophils were identified as unfavorable prognostic factors for OS and TFH and B cells as favorable prognostic factors in both cohorts (**Figure 3A**). Remarkably, high ICFR reflected better OS, regardless of whether immune cells were favorable or unfavorable prognostic factors after introducing the ICFR parameter (**Figure 3A**). This phenomenon is called the “CAF-mediated immune resistance pattern”, and we have previously reported it in abstract form (29). Among 24 types of immune cells, we selected CD8^+^ T cells as representatives of immune cells. The prognostic value of the CD8^+^ T cell/CFR had more significant power than CD8^+^ T cell and CAF alone in two independent lung cancer cohorts (**Figure 3B**), as confirmed in the multivariate analysis (**Figure 3C**).

To assess whether CFR was a prognostic factor for pan-cancer, we studied 33 independent TCGA cancer cohorts and 9,975 tumor samples with OS information. A total of 23 independent cohorts suggested that a high CFR was beneficial to OS, of which 13 cohorts [including cutaneous melanoma, urothelial bladder carcinoma, LUAD, and lung squamous cell carcinoma (LUSC)] were significantly correlated (**Figure 3D**). A total of seven cohorts (prostate adenocarcinoma, testicular germ cell tumors, pheochromocytoma and paraganglioma, thymoma, diffuse large B-cell lymphoma, cholangiocarcinoma, and uterine carcinosarcoma) demonstrated unusual confidence intervals. These were in part due to their death rates (<5% of the total) or to the small number of cohorts involved (**Supplementary Table 8**).

To translate the previous *in silico* findings in clinical samples, we analyzed the expression of CD8 and α-SMA detected by IHC in LUAD tissue microarray, including 71 LUAD tissues with overall survival data (representative sample in **Figure 3E**). α-SMA is a hallmark of the CAF activation phenotype and is encoded by ACTA2 (5, 30, 31). Seventeen of 31 TCGA solid tumor types have a striking correlation >0.70 between CAFs and ACTA2 mRNA levels (Spearman’s correlation test; LUAD: r = 0.78, 95% CI, 0.74–0.81, *p* < 0.001; **Supplementary Figure 2**). A total of 63.3% of the samples were identified as CFR^high^, according to the optimal cutoff in this validation cohort. There was a significant difference in OS between CFR^high^ and CFR^low^ groups; the median OS was 83 months (95% CI, 83 to not reachable) in patients with CFR^high^ as compared to the median OS of 22 months (95% CI, 17–38) in patients with CFR^low^ (HR, 0.21; 95% CI, 0.12–0.37; *p* < 0.001; **Figure 3F**). The CFR predicted a much higher difference in survival than those observed with CD8 or α-SMA expression alone.

### CD8^+^ T cell/cancer-associated fibroblast ratio predicts immunotherapeutic benefits across multiple cancer types

**Figure 4:**
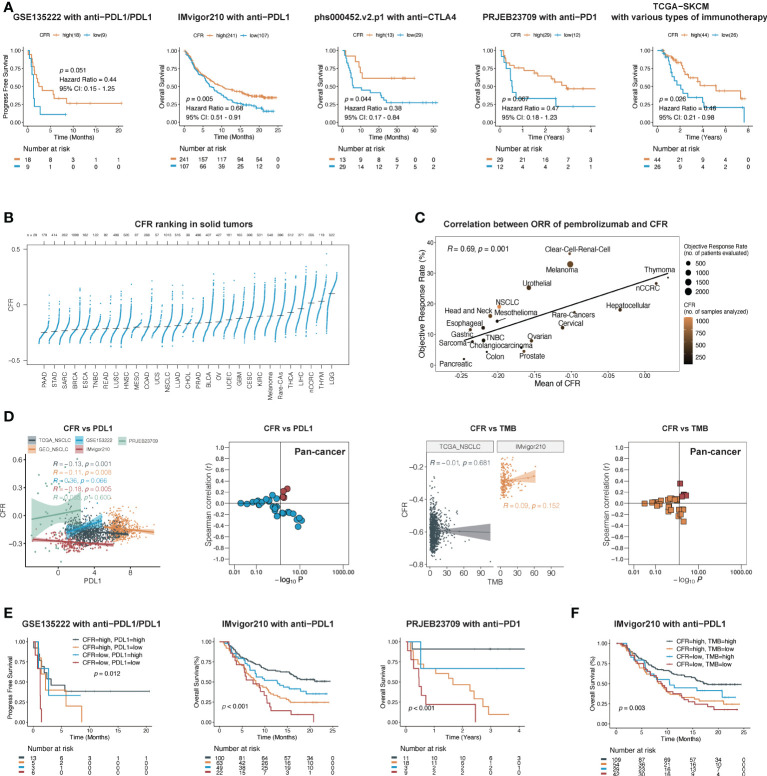
CFR is a predictive biomarker of immunotherapy across multiple cancers. **(A)** In five independent cohorts, Kaplan–Meier survival curves compare overall survival between CFR^high^ and CFR^low^ groups. **(B)** Distribution pattern of CFR in pan-cancer. Tumors are ordered by the median CFR from highest to lowest. **(C)** A correlation between CFR and objective response rate with pembrolizumab therapy in 20 solid tumor types. nCCRC, non-clear cell renal carcinoma; NSCLC, non-small cell lung cancer; rare tumors include adrenocortical carcinomas, paraganglioma-pheochromocytoma, and germ cell tumors. **(D)** A correlation between PD-L1 mRNA expression, TMB, and CFR in independent cohorts. **(E, F)** Kaplan–Meier curves of OS according to CFR combined with PD-L1 in a cohort or TMB in a cohort, respectively.

We next evaluated the predictive ability of CFR in the efficacy of immunotherapy. Five publicly available ICI datasets, including IMvigor210, PRJEB23709, phs000452.v2.p1, GSE135222, and TCGA-SKCM, were compiled for analysis. Patients with CFR^low^ tumors exhibited a worse outcome compared with those with CFR^high^ tumors, IMvigor210 cohort with PD-L1 inhibitor in urothelial carcinoma (HR, 0.68; 95% CI, 0.51–0.91; *p* = 0.005), phs000452.v2.p1 cohort with CTLA4 inhibitor in melanoma (HR, 0.47; 95% CI, 0.18–1.23; *p* = 0.067), PRJEB23709 cohort with PD-1 inhibitor in melanoma (HR, 0.38; 95% CI, 0.17–0.84; *p* = 0.044), GSE135222 cohort with PD-1/PD-L1 inhibitors in lung cancer (HR, 0.44; 95% CI, 0.15–1.25; *p* = 0.051), and TCGA-SKCM with multiple immunotherapy types in melanoma (HR, 0.46; 95% CI, 0.21–0.98; *p* = 0.026) (**Figure 4A** **)**. Three of the five immunized cohorts had Response Evaluation Criteria in Solid Tumors (RECIST) records. Among the patients in the IMvigor210 cohort, a high CFR is associated with a significant improvement in objective response rate (ORR) (30 *vs.* 10%, *p* = 0.008, **Supplementary Figure 3**). Among the patients in the PRJEB23709 cohort, a high CFR was associated with a higher ORR, which did not reach the required significance level (51 *vs.* 33%, *p* = 0.747, **Supplementary Figure 3**). A significant correlation was observed between a higher CFR and a longer overall survival term for patients in the phs000452 cohort (31 *vs.* 4%, *p* = 0.047, **Supplementary Figure 3**).

To explore the relationships between CFR and the immunotherapy responses across multiple cancer types, we identified 20 tumor types or subtypes with data regarding the ORR in unselected patients with pembrolizumab monotherapy through an in-depth literature search. Tumor types were ranked from low to high by the mean CFR (n=29, **Figure 4B**). We then plotted the ORR for anti-PD-1 or anti-PD-L1 therapy against the corresponding mean CFR (n=20, **Figure 4C**). We observed a significant correlation with a correlation coefficient of 0.69 between the CFR and the ORR (95% CI, 0.32–0.88; *p* = 0.001) (**Figure 4C**). Additionally, we analyzed the correlation between tumor mutational burden (TMB), PD-L1 expression, and immunotherapy ORR in various types of cancer. Compared with the TMB and PD-L1 expression, CFR showed an improved correlation with immunotherapy ORR (Spearman’s r = 0.69 *vs.* 0.44 and 0.21) (**Supplementary Figure 4**).

### CD8^+^ T cell/cancer-associated fibroblast ratio as an independent and complementary biomarker to programmed death-ligand 1 and tumor mutational burden

Research on PD-L1 and TMB as biomarkers of immunotherapy efficacy was the most in-depth. Therefore, we further determined the relationship between these two biomarkers and CFR. In TCGA and GEO cohorts, there was no clear evidence for a positive association between PD-L1 mRNA and CFR (Spearman’s correlation test; TCGA: r = −0.13, 95% CI, −0.19 to −0.07, *p* = 0.001; GEO: r = −0.11, 95% CI, −0.18 to −0.02, *p* = 0.008), as was further confirmed in IMvigor210, PRJEB23709, and most cohorts in pan-cancer (**Figure 4D**). In the cohorts with TMB information, the association was close to zero and not statistically significant between TMB and CFR (**Figure 4D**).

Next, we investigated whether CFR, PD-L1 expression, and TMB had an overlapping effect on the efficacy of PD-1/PD-L1 inhibitors. Each predictor showed a significant correlation with OS. With the use of both predictors and their interaction in Kaplan–Meier survival analysis, this complete model performed better than either alone. Patients with a high expression of CFR and PD-L1 had a significantly higher OS than those with a low expression of both of them in the IMvigor210 cohort (log-rank test; overall *p* < 0.001; CFR^high^ and PD-L1^high^ *vs.* CFR^low^ and PD-L1^low^: HR, 0.31; 95% CI, 0.15–0.66, *p* < 0.001) and PRJEB23709 cohort (log-rank test; overall *p* < 0.001; CFR^high^ and PD-L1^high^ *vs.* CFR^low^ and PD-L1^low^: HR, 0.06; 95% CI, 0.02–0.25, *p* < 0.001), respectively (**Figure 4E**), as did the combination with CFR and TMB in the IMvigor210 cohort (log-rank test; overall *p* < 0.001; CFR^high^ and TMB^high^ *vs.* CFR^low^ and TMB^low^: HR, 0.48; 95% CI, 0.29–0.81, *p* < 0.001; **Figure 4F**). Overall, our findings imply that CFR may play a role in patient selection in the clinic based on the unique immune microenvironment of the tumor.

### CD8^+^ T cell/cancer-associated fibroblast ratio detected by immunohistochemistry predicts PD-1/programmed death-ligand 1 inhibitor response of non-small cell lung cancer in Fujian cancer hospital cohort

**Figure 5:**
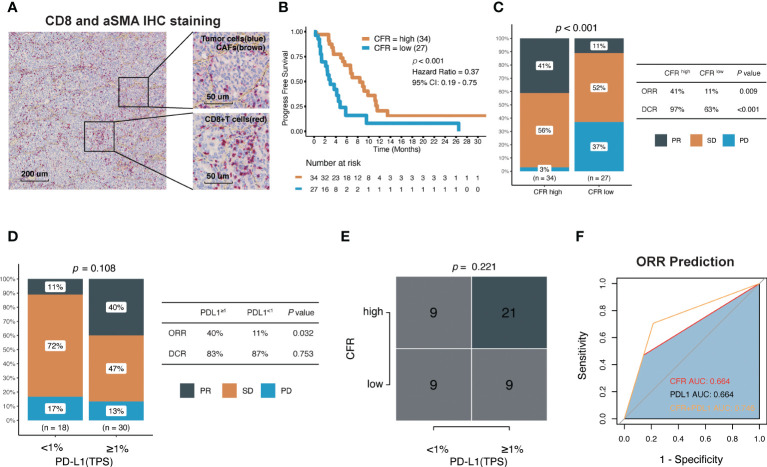
Association between CFR and clinical response to immune checkpoint inhibitors in Fujian Cancer Hospital cohort. **(A)** Representative images show double staining for α-SMA (brown) and CD8 (red). **(B)** Kaplan–Meier survival curves of PFS comparing CFR^high^ group and CFR^low^ group. **(C)** The ratio of patients with CR, PR, SD, and PD treated with anti-PD-(L)1 antibody in CFR^high^ and CFR^low^ group by chi-square test. **(D)** The ratio of patients with CR, PR, SD, and PD treated with anti-PD-(L)1 antibody in TPS ≥1% and TPS <1% using chi-square test. **(E)** A correlation between CFR and PD-L1 using chi-square test. **(F)** Receiver operating characteristic (ROC) analyses indicated that CFR plus PD-L1 (logistic regression model) harbored the highest area under the curve (AUC) (AUC = 0.746) compared with CFR and PD-L1 in NSCLC (AUC = 0.664 and 0.664, respectively). PD, progressed disease; PR, partial response; SD, stable disease; TPS, tumor proportion score.

The successful clinical translation of cancer therapeutics was facilitated by IHC biomarkers. We further confirmed the direct association of CFR detected by IHC with outcomes following anti-PD-1/PD-L1 immunotherapy in a retrospective and independent cohort of 61 patients with advanced NSCLC in FCHC. The baseline characteristics are shown in **Table 1** and **Supplementary Table 4**. Representative images indicate double staining for α-SMA and CD8 in **Figure 5A**. The median age was 61 (range, 31–79) years, and 54 patients (72%) were male. Received monotherapy and combination therapies were 19 (31%) and 42 (69%), respectively. Most patients were treated with pembrolizumab (19, 31%). The ORR was 27.9% in the entire population, and the median PFS was 5.60 months (95% CI, 4.17–9.17).

**Table 1:** Baseline characteristics of patients with advanced non-small cell lung cancer in Fujian Cancer Hospital Cohort.

	Total
	(N = 61)
**Age (years)**	61 (31–79)
**Sex**	
Female	17 (28%)
Male	44 (72%)
**Histology**	
Adenocarcinoma	27 (44%)
Squamous cell carcinoma	32 (53%)
Adenosquamous carcinoma	1 (2%)
Non-small cell lung cancer	1 (2%)
**Race**	
Asian	61 (100%)
**Type of specimens**	
Paraffin section	10 (16%)
Biopsy	51 (84%)
**Regimen**	
Combination	42 (69%)
Monotherapy	19 (31%)
**Type of checkpoint inhibitors**	
Pembrolizumab	19 (31%)
Camrelizumab	13 (21%)
Nivolumab	9 (15%)
Sintilimab	8 (13%)
Tislelizumab	5 (8%)
Others	7 (12%)
**Number of previous therapies**	
0	23 (37.7%)
1	16 (26.2%)
2	4 (6.6%)
>2	18 (29.5%)
**PD-L1**	
TPS < 1%	18 (29.5%)
TPS ≥ 1%	30 (49.2%)
Unknown^*^	13 (21.3%)
**Response**	
PD	11 (18.0%)
PR	17 (27.9%)
SD	33 (54.1%)

TPS, tumor proportion score; CR, complete response; PR, partial response; SD, stable disease; PD, progressive disease.

^*^No records in hospital pathology system.

The median PFS of CFR^high^ *vs.* CFR^low^ was 8.37 *vs.* 2.97 months (HR, 0.37; 95% CI, 0.19–0.75; *p* < 0.001; **Figure 5B**). In terms of efficacy, the response evaluation according to RECIST1.1 in CFR^high^ tumors was partial response (PR) 41%, stable disease (SD) 56%, and progressive disease (PD) 3% compared with PR 11%, SD 52%, and PD 37% in CFR^low^ tumors (*p* < 0.001; **Figure 5C**), respectively. Patients with CFR^high^ had higher ORR (41% *vs.* 11%; chi-square test, *p* = 0.009; **Figure 5C**) and higher disease control rate (DCR) (97% *vs.* 63%; chi-square test, *p* < 0.001; **Figure 5C**) compared with CFR^low^.

Among the 48 patients with documented PD-L1 expression, 18 (38%) had TPS ≥1%, and 23% (62%) had TPS <1% (**Table 1**). The effect of the PD-L1 expression level on PFS (HR, 0.9; 95% CI, 0.43–1.86; *p* = 0.775) and DCR was not obvious, but the effect on ORR was significant. Patients with TPS ≥1% had higher ORR (40% *vs.* 11%; chi-square test, *p* = 0.032; **Figure 5D**). Although the proportion of patients with TPS ≥1% was higher in high CFR, no statistical significance between these two groups was observed (chi-square test, *p* = 0.221; **Figure 5E**). Surprisingly, CFR plus PD-L1 (AUC = 0.764) was superior to either CFR (AUC = 0.664) or PD-L1 (AUC = 0.664) in predicting ORR **(** **Figure 5F**).

### Comparison of the performance of the CD8^+^ T cell/cancer-associated fibroblast ratio model with a four-class classification model and immunophenotype

We further compared the predictive performance of the CFR model with that of a four-class classification model in the IMvigor210 cohort with the largest sample size. This model is based on the optimal cutoff values for distinguishing OS of CD8^+^ T cells and CAFs, which divided the patients into four subgroups: CD8^high^CAFs^high^, CD8^low^CAFs^high^, CD8^high^CAFs^low^, and CD8^low^CAFs^low^. The difference in OS between CFR^high^ and CFR^low^ predicted by the CFR model was significantly higher than that between the four groups predicted by the classification model (**Supplementary Figure 6A**). Additionally, the predictions of ORR produced by the CFR model were more significant for statistics than the four-class classification model (**Supplementary Figure 6B**). Notably, the CD8^high^CAFs^low^ group significantly outperformed the other three groups in terms of OS and ORR and even surpassed the CFR^high^ group, but the number of patients is considerably lower. In addition, the rate of clinical response did not differ between the CD8^high^CAFs^high^ and CD8^low^CAFs^low^ groups. Overall, the dichotomous model outpredicted a four-class classification model in terms of overall statistical and clinical significance. We also analyzed the immunohistochemical cohort and obtained similar results; no statistically significant difference in prognostic curves was demonstrated between the CD8^high^CAFs^high^ and CD8^low^CAFs^low^ subgroups based on the four-class classification model (**Supplementary Figure 6C**). The abundance of intratumoral CAFs and CD8 T-cell infiltration was classified into four classes observed in the immunohistochemical samples (**Supplementary Figure 5**).

Most solid tumors were classified into three distinct immune phenotypes in the TME: the inflamed phenotype, immune-excluded phenotype, or immune-desert phenotype. There were no statistically significant differences among these three immunotypes for OS in the IMvigor210 cohort (overall *p* = 0.090; inflamed: **Supplementary Figure 6D**). More specifically, patients with inflamed immunotypes showed the best prognosis (inflamed *vs.* others: HR, 0.69, 95% CI, 0.51–0.95, *p* = 0.033), and those with excluded or desert immunotypes showed a similar prognosis (excluded *vs.* desert: HR, 0.91, 95% CI, 0.65–1.28, *p* = 0.594). Among the three groups, there were no statistically significant differences in CFR (Kruskal–Wallis test, overall *p* = 0.087; **Supplementary Figure 6E**). Furthermore, it was found that CFR enhanced OS in the inflamed and excluded groups (HR, 0.57, 95% CI, 0.26–1.25; *p* = 0.096 and HR, 0.55, 95% CI, 0.35–0.85; *p* = 0.003, respectively, **Supplementary Figure 6F**), but not the OS in the desert group (HR, 0.94, 95% CI, 0.52–1.70; *p* = 0.840, **Supplementary Figure 6F**).

### Exploration of the differences in molecular characteristics of the CD8^+^ T cell/cancer-associated fibroblast ratio subgroup

**Figure 6:**
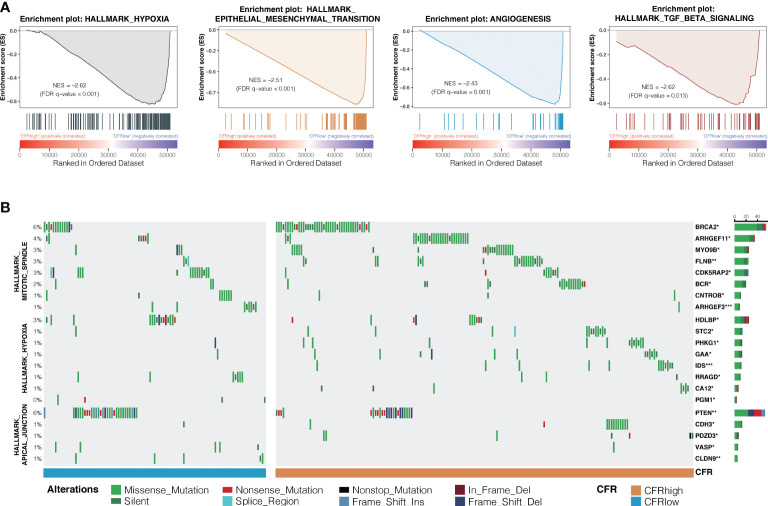
Differences in signaling pathways and genes between CFR subgroups. **(A)** Prominent enrichments of signatures in CFR^high^ *vs.* CFR^low^ groups. FDR, false discovery rate. **(B)** Mutation enrichment characteristics among subgroups. A group of genes significantly more mutated than the other groups are displayed (Fisher’s test, *p < 0.05, **p < 0.01, ***, *p* < 0.001) display the top 20 most significant genes.

Hallmark gene sets based on the RNA and WES data from TCGA database were analyzed to investigate the underlying mechanism of CFR’s prognostic and predictive values in ICI efficacy. GSEA in NSCLCs revealed a prominent enrichment of signatures related to the upregulation of hypoxia, TGF-β signaling, epithelial–mesenchymal transition, and angiogenesis in the CFR^high^ *vs.* CFR^low^ groups (FDR adjusted *p* < 0.05 for all; **Figure 6A**). We also analyzed the significant difference in gene mutation characteristics between the two subgroups and selected the top 20 mutated genes involved in the Apical Junction, Hypoxia, and Mitotic spindle pathway (Fisher’s test, *p* < 0.05; **Figure 6B**). Finally, the tumor immune dysfunction and exclusion (TIDE) (32) algorithm was used to investigate the relationship between TIDE and CFR. We found that the TIDE score of the CFR^low^ group was higher than that of the CFR^high^ group (Wilcoxon test, *p* < 0.001, **Supplementary Figure 6G**). The above results strongly suggest that the tumor immune escape mechanism was enhanced in the CFR^low^ group.

## Discussion

We precisely assessed the immune response status of the TME by calculating the potential negative effect of CAFs, thereby generating a novel parameter deemed CFR. The findings show that the novelty parameter CFR can stratify patients with metastatic cancer into groups with different prognoses and clinical responses to ICI treatment across multiple cancer types in different dataset platforms. These results demonstrate the potential clinical utility of CFR, at either a transcriptional level or the protein immunohistochemistry level. Therefore, CFR is a simple, newly found, clinically applicable parameter that can be useful in identifying patients unlikely to benefit from immunotherapy.

The major finding in this study is that CAFs were moderately positively correlated with most immune cell populations, irrespective of their type. The current consensus is that the predominant effect of CAFs is immunosuppression, collaboration with IL-6, CXC-chemokine ligand 9 (CXCL9), and TGF-β, demonstrating well-established roles in recruiting and activating the myeloid-derived suppressor cells (MDSCs), Treg, and tumor-associated neutrophils and reducing CD8^+^ T-cell recruitment and responses (4, 5). Nevertheless, the relationship between CAFs and immune cells varies with tumor purity and stromal- or immune-dominant pattern due to spatial heterogeneity in the TME. *In vivo*, Ene-Obong et al. characterized the immune infiltration within stromal sub-compartments of pancreatic ductal adenocarcinoma and identified that cytotoxic effector T cells (CD8^+^) were significantly reduced in the juxtatumoral compartments compared to the pan-stromal areas (33). Coincidentally, Kato et al. found spatial distributions of the significant correlation between CAFs and CD8^+^ T cells in esophageal cancer tissues with immunofluorescence imaging. In cases where CAFs are present in high numbers, CD8^+^ TILs in the intratumoral tissues were scarce, despite an accumulation of CD8^+^ T cells in peritumoral sites (34). Ultimately, CAFs influence the spatial distribution of immune cells, but the impact of CAFs on the total amount of distinct immune cell infiltration has not yet been well-established. Therefore, compared with the local assessment, comprehensive quantitative results based on bulk tumor samples RNA-seq could better reflect the panorama of the intricate interplay of CAFs with the status of the whole immunocyte infiltration. In this study, Th17 was negatively associated with CAFs in many cancers including lung cancer, implying that it could be beneficial for antitumor immune responses. However, considering that Th17 is induced by TGF-β and is a subset that promotes tumor growth (35), despite priming cytotoxic T cells with antitumor activity and favorable prognosis (36, 37), their role in cancer remains controversial (38). Alternatively, the difference in the design of the Th17 signature is a factor that may have contributed to the different outcomes.

Previous signatures that did not consider stromal components classified the TME into three subtypes, primarily differing by inflammation level (39, 40). Relying solely on the assessment of immune cells, infiltration would be insufficient to evaluate the complexity of the TME accurately. In this study, conflicting findings on the association between distinct immune cell infiltration and OS in various datasets illustrate the methodological limitations of using single-parameter assessment. Additionally, patients with a similar immunophenotype have a heterogeneous prognosis using CFR as a stratification factor. The bivariate model has proved more powerful as a predictive biomarker than either single variable model alone. Several attempts have been made to explore the joint effect of CAFs and immune cells in cancer. CAFs coexisting with Tregs (41), M2 macrophages (42, 43), and CD8^+^ T cells (17) were correlated with a poor outcome in cancer. However, in these studies, CAFs and immune cells were included in the model as categorical covariates, posing considerable beneficial information loss. Driven by machine learning (44, 45) and mechanism-based insights (46), we developed the immune cell/CAF ratio as a quantitative assessment of the interaction of immune cells and CAFs. Our model formulation and implementation offer several important advantages. First, ICFR became a protective prognostic factor when ICFR was introduced, regardless of specific immune cell types.

Moreover, a stronger prognostic performance was observed in the CFR than in the CD8^+^ T cells. Second, the CFR model displayed a better predictive value than the four-class classification model with categorical covariates. More importantly, this characterized feedback regulation between CAFs and immune status highlights that a high degree of immune cell infiltration does not imply high immunotherapy efficacy, along with abundant CAFs. In the same manner, low immune cell infiltration accompanied by a low number of CAFs did not guarantee the poor therapeutic efficacy of immunotherapy. In line with our results, Bagaev et al. recently identified four distinct microenvironments termed immune-enriched, non-fibrotic (IE), immune-enriched, fibrotic (IE/F), immune-depleted (D), and fibrotic (F) sorted from high to low immunotherapy response across diverse cancers (47).

Recently, predictive immunotherapy biomarkers have received increasing attention. To date, PD-L1 expression and TMB are the most thoroughly investigated in the literature (48). Our findings highlight CFR as a promising predictive biomarker that could be significantly superior to PD-L1, TMB, or their combination. Recent studies supported PD-L1 expression as a potential biomarker for pembrolizumab in NSCLC (23, 49, 50). However, its predictive value in other cancer types or anti-PD-1 drugs remains controversial (51). Although TMB-High serves as a pan-cancer biomarker of pembrolizumab treatment approved by the FDA (52, 53), TMB faces numerous challenges (54, 55). A recent study found that high TMB fails to predict immune checkpoint blockade response across all cancer types (56). Compared with low TMB, high TMB tumors only showed an improved response rate in cancer types where CD8 T-cell levels were positively correlated with neoantigen load (57). Theoretically, CFR, PD-L1, and TMB were from different biological sources in response to ICI, indicating the immune microenvironment and tumor aspect. Data from this study confirm that CFR and PD-L1 expression and TMB are independent biomarkers with a largely additive ability to predict benefits from immune checkpoint inhibition. Patients with high CFR, high PD-L1 expression, or high TMB had significantly improved OS with ICI therapy compared with patients whose tumors had only one or neither variable. Disease-specific CFR thresholds warrant further investigation into various malignancies.

This research has several limitations. First, several research findings established the phenotypic and functional diversity of CAFs (58, 59) and immune cell populations, especially intratumoral CD8^+^ T cell (60–62). Therefore, the interaction between various CAF subtypes with immune cell subtypes and their optimal combination must be further explored. Second, the efficacy of ICI was shown to be dependent on the cancer being an immunogenic “hot” tumor and not a hypoimmunogenic “cold” tumor (63). Indeed, employing the model with T cells as the major parameter to identify potential ICI beneficial populations in the immune desert subgroup is difficult. Third, as every coin has two sides, we developed a practical and straightforward method using CD8^+^ T cell/CAF field area ratio by IHC to evaluate their interplay in the clinical validation cohort. However, the entire study may be insufficient to transfer to the sole use of α-SMA, although we found a striking correlation >0.70 between the two variables in 17 TCGA solid tumor types, providing compelling evidence of their tight relation. Meanwhile, IHC for other markers (e.g., FAP, CD29, PDGFRβ, S100A4, and FSP1) of CAF may be a useful biomarker in addition to α-SMA in which to select patients. The fourth limitation is the potential for confounding, especially with respect to the predictive significance. Across the immunotherapy-treated cohort, we lacked information on potentially important confounders. Also, in the absence of a control arm, CFR may be prognostic, and future controlled studies are needed to confirm a potential predictive role. Finally, the model in this study did not consider cancer cell factors, although genomic differences were analyzed. Further efforts to integrate with genomic alterations may potentially enhance patient stratification.

In conclusion, this study developed a novel parameter to evaluate the immune response status of the TME more precisely by analyzing the effect of the presence of CAFs on CD8^+^ T cells. Furthermore, CAF and immune cells can jointly stratify metastatic cancer types into subgroups with different prognoses and heterogeneous clinical responses to ICI treatments. These factors may guide immunotherapeutic decisions for patients with metastatic cancers, especially in the context of NSCLC.

## Data availability statement

The original contributions presented in the study are included in the article/**Supplementary Material**. Further inquiries can be directed to the corresponding authors.

## Ethics statement

The studies involving human participants were reviewed and approved by Ethics Committee of Fujian Cancer Hospital. The patients/participants provided their written informed consent to participate in this study. Written informed consent was obtained from the individual(s) for the publication of any potentially identifiable images or data included in this article.

## Author contributions

XLZ and KJ: data curation, formal analysis, investigation, methodology, writing—original draft, and project administration. WX, DZ, WP, XC and PL: data curation, formal analysis, and investigation. LZ, XBZ, QM, HW, SW, YX, HX, CL, LL, XG, SZ and JL: resources, formal analysis, and investigation. DW and ZZ: methodology. SY: formal analysis. YL and ZC: methodology. QZ, LC and JB: data curation and methodology. XX: resources. XL and GL: conceptualization, resources, data curation, formal analysis, supervision, funding acquisition, validation, investigation, methodology, project administration, and writing—review and editing. All authors contributed to the article and approved the submitted version.

## Acknowledgments

We thank the researchers at the Geneplus-Beijing Institute for supporting the bioinformatics analysis of this study and Tony Mok of the Chinese University of Hong Kong for critical reading of the manuscript. This work was supported by the National Natural Science Foundation of China (grant 82072565), the Fujian Provincial Science and Technology Department guided projects (grant 2020Y9038), the Fujian Provincial Health Systemic Innovation Project (grant 2020CXA010), the Bejing Xisike Clinical Oncology Research Foundation (grant Y-2019AZZD-0386), and the Fujian Provincial Clinical Research Center for Cancer Radiotherapy and Immunotherapy (grant 2020Y2012).

## Conflict of interest

The authors declare that the research was conducted in the absence of any commercial or financial relationships that could be construed as a potential conflict of interest.

## Publisher’s note

All claims expressed in this article are solely those of the authors and do not necessarily represent those of their affiliated organizations, or those of the publisher, the editors and the reviewers. Any product that may be evaluated in this article, or claim that may be made by its manufacturer, is not guaranteed or endorsed by the publisher.
