# Supplementary material for: CD8+ T cell/cancer-associated fibroblast ratio stratifies prognostic and predictive responses to immunotherapy across multiple cancer types
Source: Front Immunol. 2022 Nov 9;13:974265. doi: 10.3389/fimmu.2022.974265 (PMC9682254; doi:10.3389/fimmu.2022.974265)
Supplement: Supplementary file 1 [file DataSheet_1.pdf]

Supplementary Figures

CD8+ T Cell/Cancer-Associated Fibroblasts Ratio Stratifies Prognostic and Predictive Responses to Immunotherapy across Multiple Cancer Types

Supplementary Figure 1. CAFs are the dominant cell type over immune cells in the tumor microenvironment

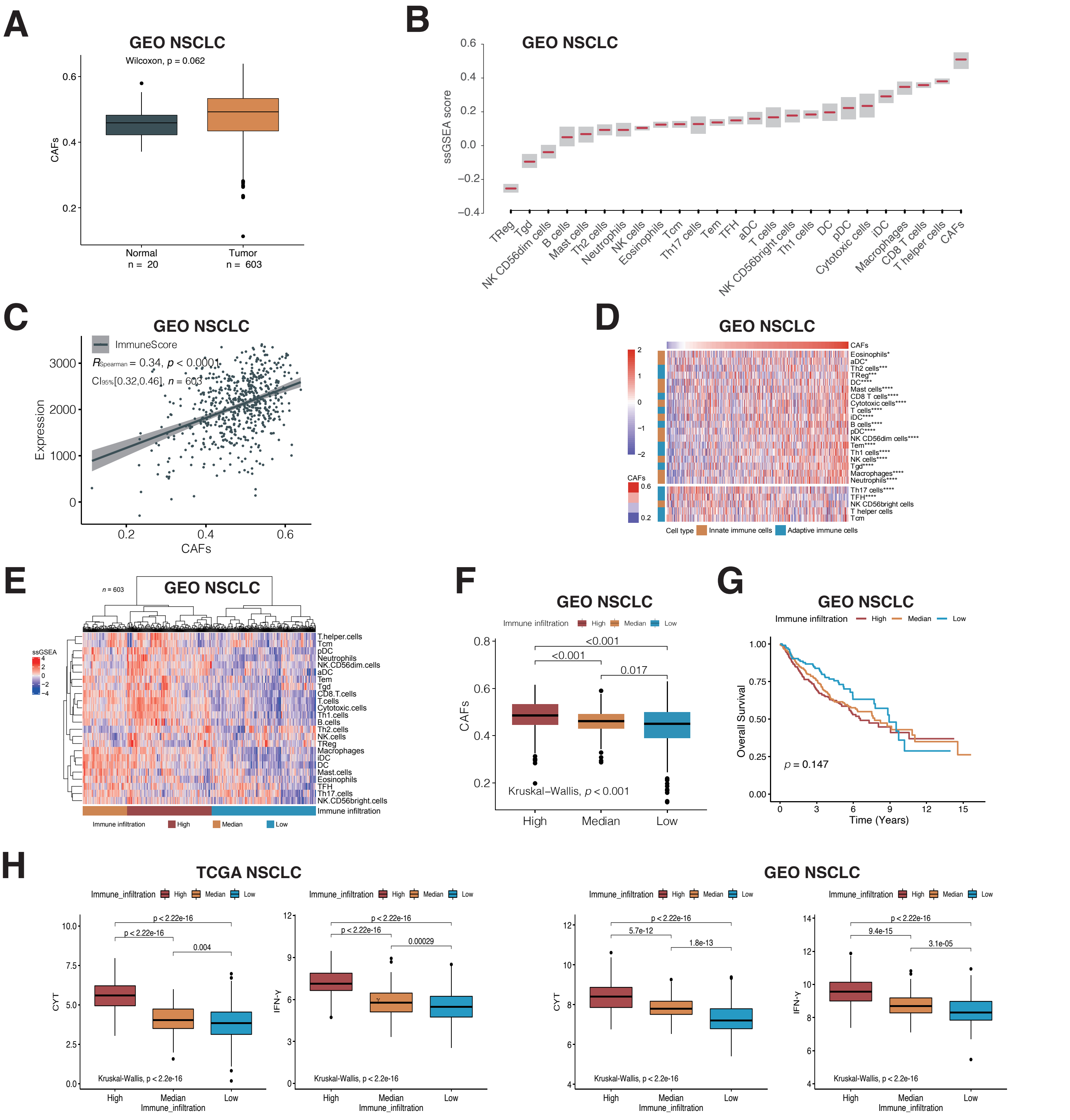

A. Analysis of the GEO cohorts found that cancer-associated fibroblasts (CAFs) were significantly higher in tumor tissues than in normal (median value, 0.466 vs. 0.447). **B.** The relative amounts of CAFs and different immune cell types in the GEO cohort. The lower and upper bounds of the box in a boxplot indicate the first and third quartiles, respectively. Cell types are ordered by their median ssGSEA score. **C.** Correlation between CAFs and ImmuneScore in GEO cohorts.**D.** The non-clustering heat map is arranged according to the correlation coefficient between CAF and 24 types of immune cells in GEO cohorts. The colors from red to blue denoted their correlation coefficients of the immune score and CAFs from high to low. The upper part indicates immune cells that are positively correlated with the content of fibroblasts, and the lower part shows negative correlations. *, p <0.05; **, p <0.01; ***, p <0.001.**E.** Unsupervised clustering of TME cells for 603 patients in the GEO cohort. F. The CAF difference analysis between high, medium, and low tumors with immune infiltration in GEO cohorts. **F.** The CAF difference analysis between high, medium, and low tumors with immune infiltration in GEO cohorts (median value; 7.74, 8.93 , 6.56). **G.** Differences in Overall Survival among the three immune infiltration levels were analyzed in the GEO cohorts. **H.** Differences in CYT, and INF-γ among the three immune infiltration levels were analyzed in the TCGA and GEO cohorts.CYT: Cytolytic Activity.

Supplementary Figure 2. Correlation between ACTA2 and CAFs in 31 TCGA solid cancer types

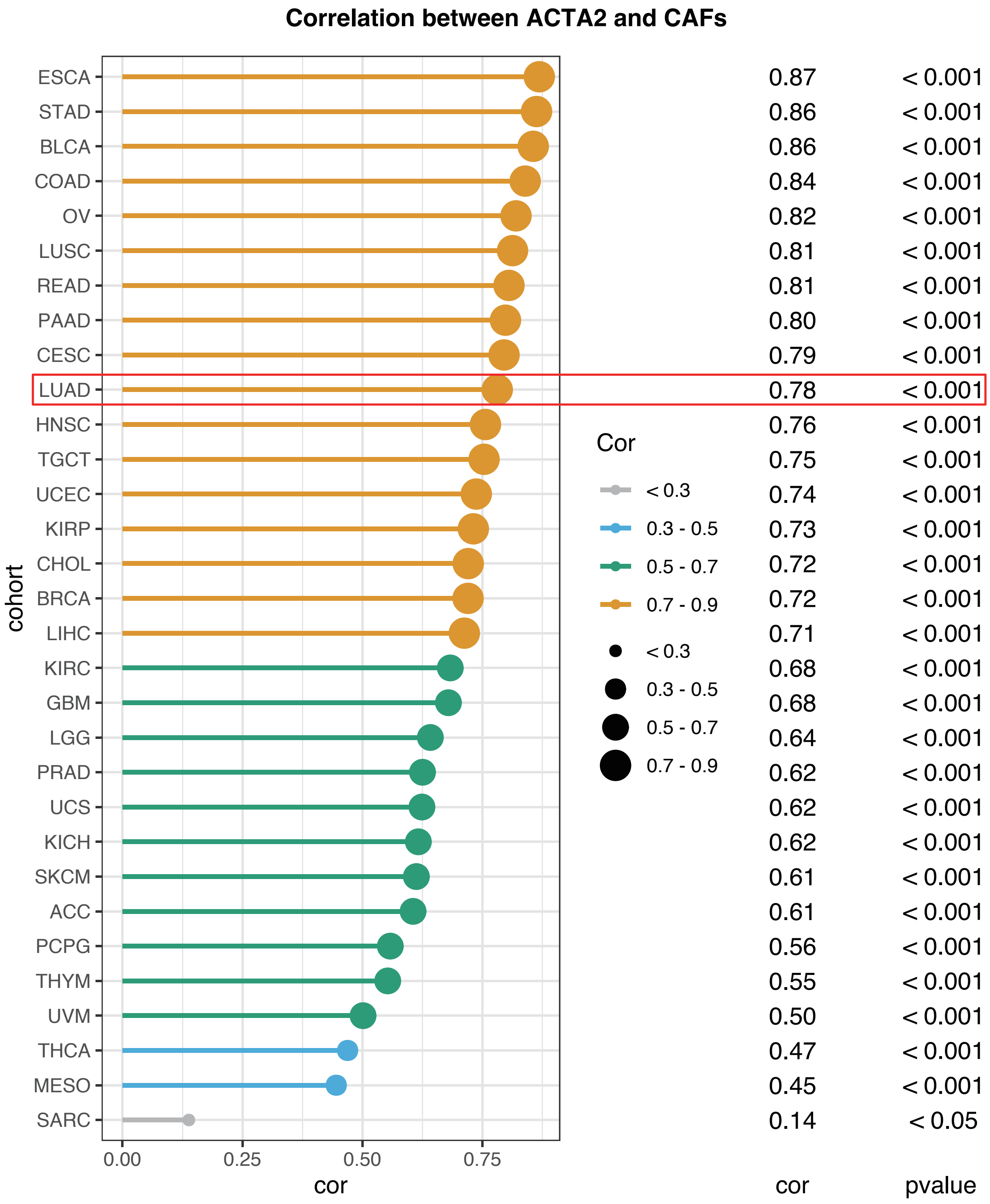

Correlation analysis of ACTA2 (also known as α-SMA) mRNA levels and CAFs in 31 TCGA solid cancer types. Results are displayed as a 'lollipop' chart where color , length of the lollipop indicates log2 fold change (X-axis), and size of the lollipop all reflects spearman 's correlation coefficient.

Supplementary Figure 3. Association between CFR and treatment response in three independent immunotherapy cohorts.

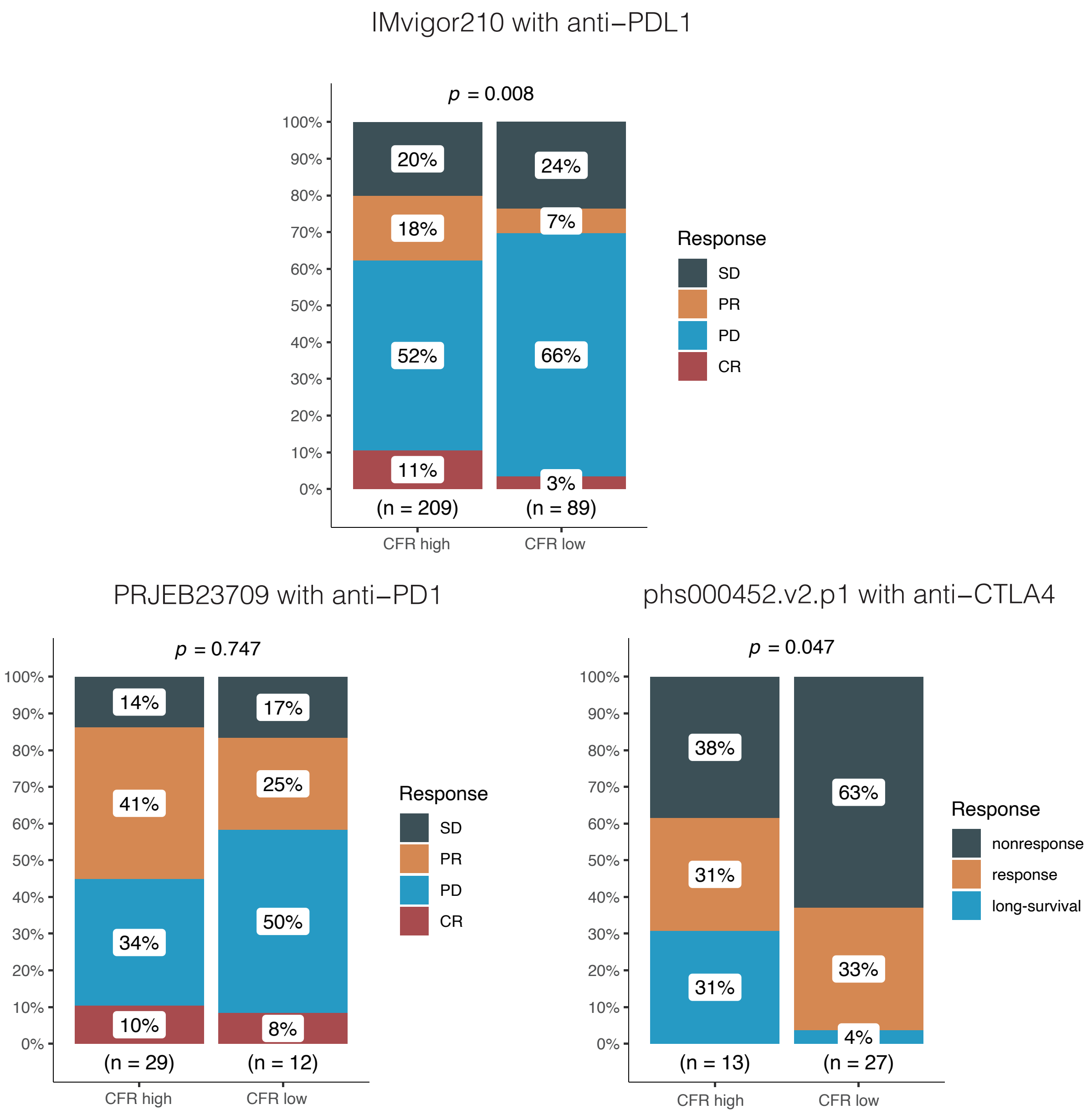

Rate of clinical response (complete response [CR], partial response [PR] , stable disease [SD], progressive disease [PD]/response, nonresponse and long-survival) to ICI in high or low CFR groups in three cohort (Chi-square test). Display cohort names and subgroup sample numbers. The evaluation criteria of treatment response were based on RECIST1.1.

Supplementary Figure 4. Analysis of PDL1 and TMB correlated with objective response rates with pembrolizumab therapy in 20 tumor types

A

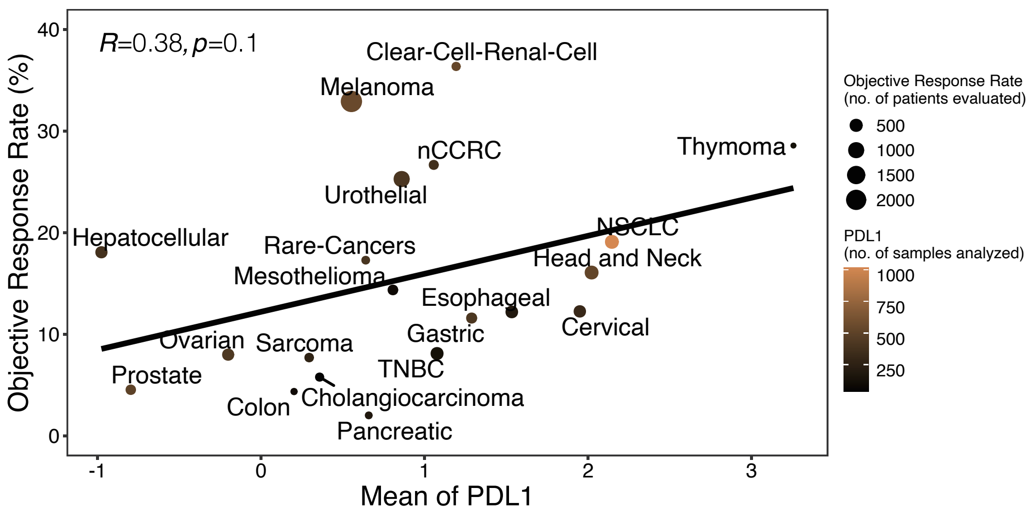

B

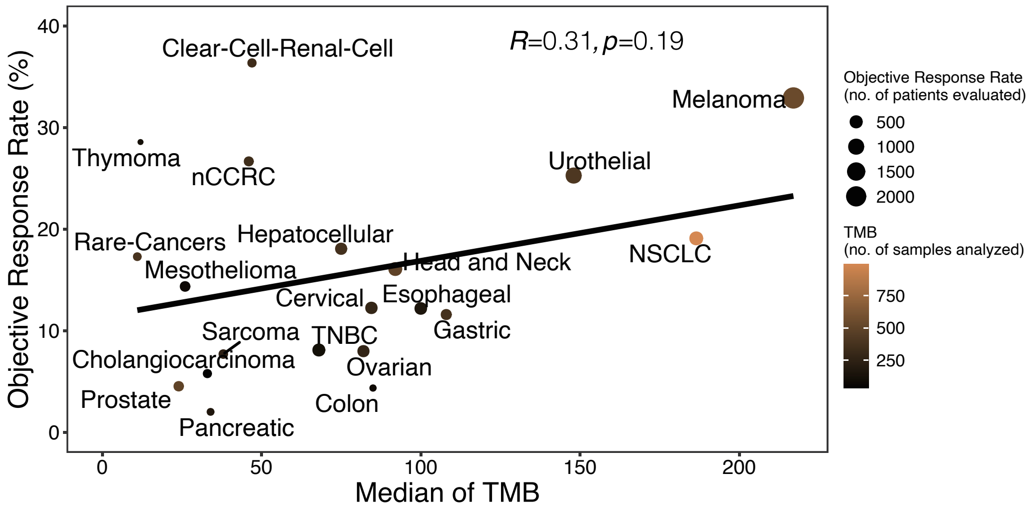

Evaluation of PD-L1 **(A)** and TMB **(B)** was obtained from the TCGA pan-cancer cohort. Pembrolizumab ORR data were obtained from published clinical studies (see appendix). The circle size represented the number of patients evaluated for the objective response rate in each tumor type, and the degree of color indicated the number of tumor samples analyzed to calculate the PD-L1 or tumor mutational burden.TMB, tumor mutational burden; nCCRC, non-clear cell renal carcinoma; NSCLC， non-small cell lung cancer; Rare tumors include adrenocortical carcinomas, paraganglioma-pheochromocytoma and germ cell tumors.

Supplementary Figure 5. Representative immunohistochemical images of the abundance of intratumoral CAF and CD8 T cell infiltration

CD8 low & CAFs high = CFR low

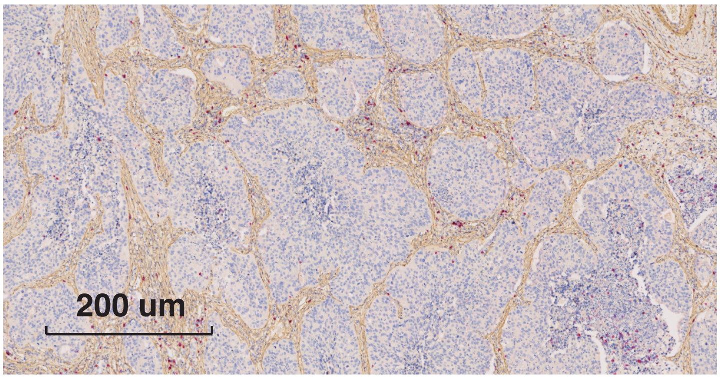

CD8 high & CAFs low = CFR high

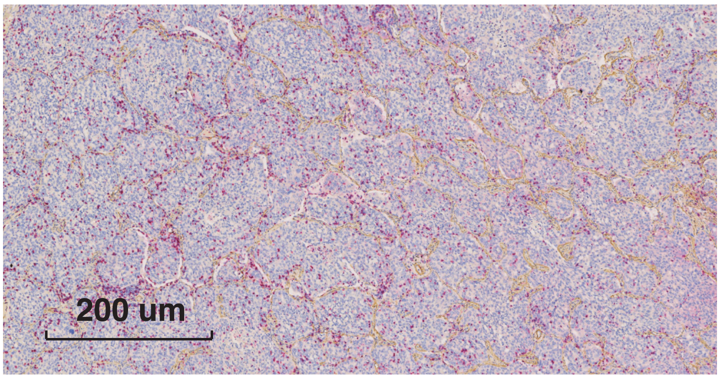

CD8 high & CAFs high

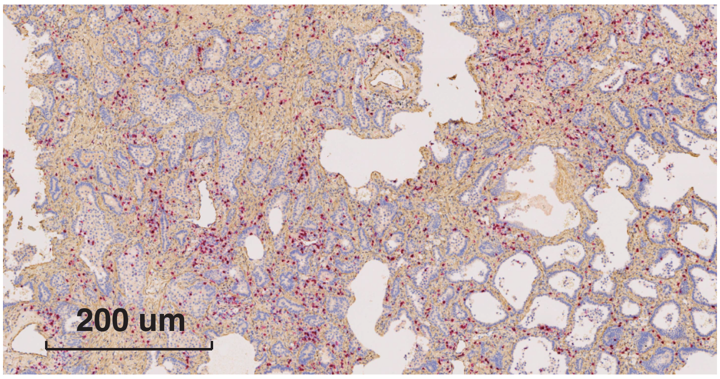

CD8 low & CAFs low

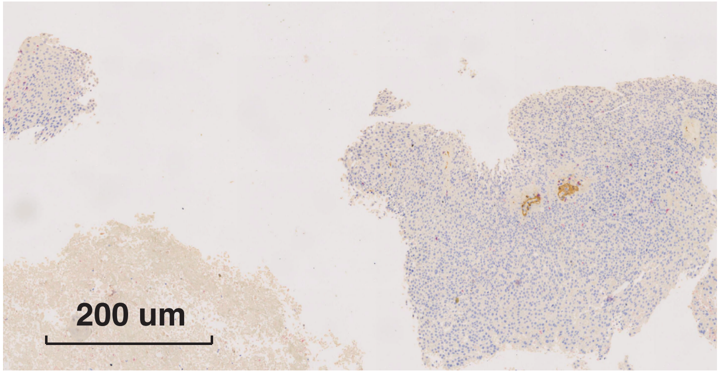

CD8+T cells(red)   Tumor cells(blue)   CAFs(brown)

Supplementary Figure 6. Comparison of the performance of CFR model with four-class classification model and immunophenotype.

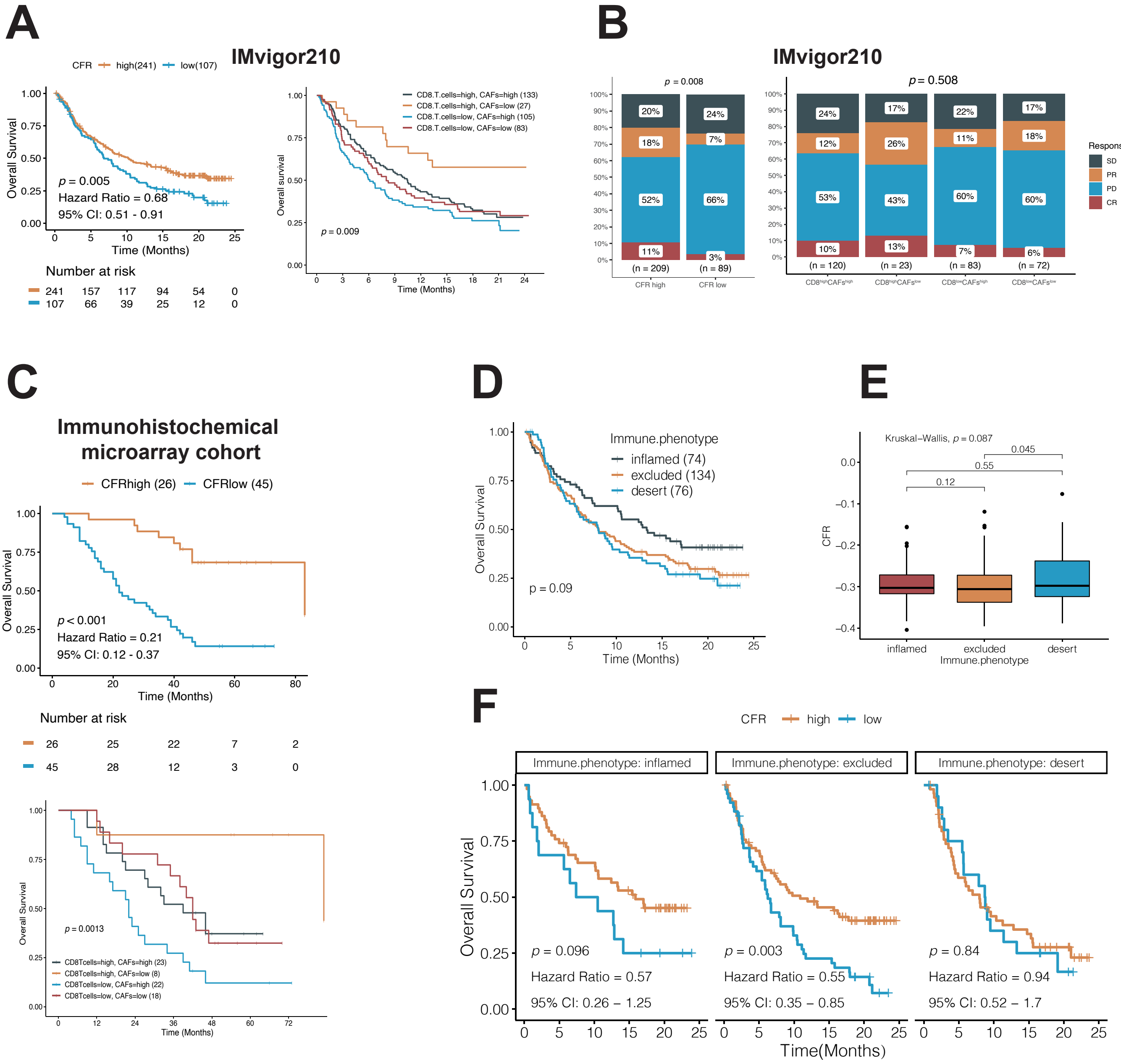

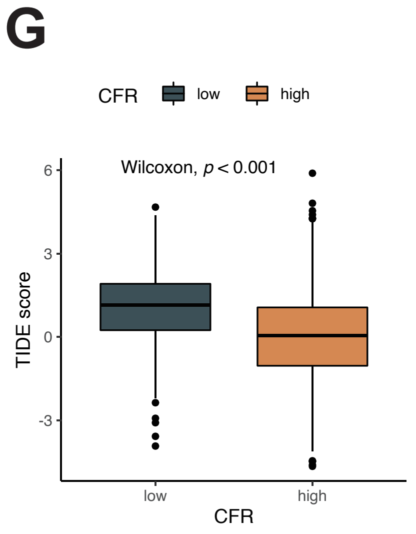

A and **C**. Left, Kaplan–Meier curves for high and low CFR patient groups; Right, Kaplan–Meier curves for the four groups of patients. Log-rank test. The cohort name is displayed at the top. Display sub-group numbers. **B**. Rate of clinical response (complete response [CR], partial response [PR] , stable disease [SD], progressive disease [PD]/response, nonresponse and long-survival) to ICI in high or low CFR groups in three cohort (Fisher exact test). Display cohort names and subgroup sample numbers. **D**. Kaplan-Meier analysis was performed to compare OS between desert, excluded, and inflamed immunotypes. **E**. Boxplot showing CFR in groups with desert (n = 76, -0.298) or excluded (n = 134, -0.306) and inflamed (n = 74, -0.303) immunophenotype. The differences between every two groups were compared through the Kruskal–Wallis test. P values indicated. **F**. Subgroup analysis of the relationship between CFR and OS in three immunotypes;**G**. Analysis of the TCGA NSCLC cohorts found that TIDE score were significantly higher CFR low (median = 1.15) than CFR high (median = 0.05) . CD8+ T cell/CAF ratio: CFR.
