# Supplementary material for: CD8+ T cell/cancer-associated fibroblast ratio stratifies prognostic and predictive responses to immunotherapy across multiple cancer types
Source: Front Immunol. 2022 Nov 9;13:974265. doi: 10.3389/fimmu.2022.974265 (PMC9682254; doi:10.3389/fimmu.2022.974265)
Supplement: Supplementary file 2 [file DataSheet_2.pdf]

Supplemental Appendix

Table S1 **..2**

Search Strategy **...3**

References **..4**

Table S1: List of citations for individual studies used in pooled analysis of objective response rate.

Cancer Type	References					
Cervical Cancer	1	2				
Clear-Cell Renal Cell Carcinoma (ccRCC)	3					
Colon	4					
Esophageal Carcinoma	5	6				
Gastric Cancer	7					
Head and Neck	8	9	10			
Hepatocellular Carcinoma	11	12				
Melanoma	13	14	15	16	17	18
Mesothelioma	19	20	21			
non-clear cell Renal Cell Carcinoma (nccRCC)	22					
Non-Small-Cell Lung (NSCLC)	23	24				
Ovarian Cancer	25					
Pancreatic Cancer	26	27				
Prostate Cancer	28					
Rare-Cancers	29	30	31	32		
Sarcoma	33	34				
Thymoma	34	35				
Triple-Negative Breast Carcinomas (TNBC)	36	37				
Urothelial Carcinoma	38	39	40	41	42	43
Biliary Cancer	44					

Search Strategy

We searched for clinical trials in NCBI using the specific search term pembrolizumab. A total of 437 results were retrieved up to December 2021. We reviewed each outcome in detail and counted only those clinical studies that met the inclusion criteria. Inclusion criteria: 1. Clinical studies with ORR data for treatment with only one agent, pembrolizumab; 2. And from non-selective populations (data from studies with only PDL1-positive patients cannot be accommodated; or data from studies with only driver-negative patients cannot be accommodated, except for triple-negative breast cancer). If multiple clinical studies were conducted for a cancer, the results of its ORR need to be aggregated for all clinical study data. Briefly, we counted ORR data for a total of 20 cancers, which were obtained from 44 references.

References

1. Chung, H. C.; Ros, W.; Delord, J. P.; Perets, R.; Italiano, A.; Shapira-Frommer, R.; Manzuk, L.; Piha-Paul, S. A.; Xu, L.; Zeigenfuss, S.; Pruitt, S. K.; Leary, A., Efficacy and Safety of Pembrolizumab in Previously Treated Advanced Cervical Cancer: Results From the Phase II KEYNOTE-158 Study. *J Clin Oncol* **2019,** *37* (17), 1470-1478.
2. Colombo, N.; Dubot, C.; Lorusso, D.; Caceres, M. V.; Hasegawa, K.; Shapira-Frommer, R.; Tewari, K. S.; Salman, P.; Hoyos Usta, E.; Yañez, E.; Gümüş, M.; Olivera Hurtado de Mendoza, M.; Samouëlian, V.; Castonguay, V.; Arkhipov, A.; Toker, S.; Li, K.; Keefe, S. M.; Monk, B. J., Pembrolizumab for Persistent, Recurrent, or Metastatic Cervical Cancer. *N Engl J Med* **2021,** *385* (20), 1856-1867.
3. McDermott, D. F.; Lee, J. L.; Bjarnason, G. A.; Larkin, J. M. G.; Gafanov, R. A.; Kochenderfer, M. D.; Jensen, N. V.; Donskov, F.; Malik, J.; Poprach, A.; Tykodi, S. S.; Alonso-Gordoa, T.; Cho, D. C.; Geertsen, P. F.; Climent Duran, M. A.; DiSimone, C.; Silverman, R. K.; Perini, R. F.; Schloss, C.; Atkins, M. B., Open-Label, Single-Arm Phase II Study of Pembrolizumab Monotherapy as First-Line Therapy in Patients With Advanced Clear Cell Renal Cell Carcinoma. *J Clin Oncol* **2021,** *39* (9), 1020-1028.
4. O'Neil, B. H.; Wallmark, J. M.; Lorente, D.; Elez, E.; Raimbourg, J.; Gomez-Roca, C.; Ejadi, S.; Piha-Paul, S. A.; Stein, M. N.; Abdul Razak, A. R.; Dotti, K.; Santoro, A.; Cohen, R. B.; Gould, M.; Saraf, S.; Stein, K.; Han, S. W., Safety and antitumor activity of the anti-PD-1 antibody pembrolizumab in patients with advanced colorectal carcinoma. *PLoS One* **2017,** *12* (12), e0189848.
5. Shah, M. A.; Kojima, T.; Hochhauser, D.; Enzinger, P.; Raimbourg, J.; Hollebecque, A.; Lordick, F.; Kim, S. B.; Tajika, M.; Kim, H. T.; Lockhart, A. C.; Arkenau, H. T.; El-Hajbi, F.; Gupta, M.; Pfeiffer, P.; Liu, Q.; Lunceford, J.; Kang, S. P.; Bhagia, P.; Kato, K., Efficacy and Safety of Pembrolizumab for Heavily Pretreated Patients With Advanced, Metastatic Adenocarcinoma or Squamous Cell Carcinoma of the Esophagus: The Phase 2 KEYNOTE-180 Study. *JAMA Oncol* **2019,** *5* (4), 546-550.
6. Kojima, T.; Shah, M. A.; Muro, K.; Francois, E.; Adenis, A.; Hsu, C. H.; Doi, T.; Moriwaki, T.; Kim, S. B.; Lee, S. H.; Bennouna, J.; Kato, K.; Shen, L.; Enzinger, P.; Qin, S. K.; Ferreira, P.; Chen, J.; Girotto, G.; de la Fouchardiere, C.; Senellart, H.; Al-Rajabi, R.; Lordick, F.; Wang, R.; Suryawanshi, S.; Bhagia, P.; Kang, S. P.; Metges, J. P., Randomized Phase III KEYNOTE-181 Study of Pembrolizumab Versus Chemotherapy in Advanced Esophageal Cancer. *J Clin Oncol* **2020,** *38* (35), 4138-4148.
7. Fuchs, C. S.; Doi, T.; Jang, R. W.; Muro, K.; Satoh, T.; Machado, M.; Sun, W.; Jalal, S. I.; Shah, M. A.; Metges, J. P.; Garrido, M.; Golan, T.; Mandala, M.; Wainberg, Z. A.; Catenacci, D. V.; Ohtsu, A.; Shitara, K.; Geva, R.; Bleeker, J.; Ko, A. H.; Ku, G.; Philip, P.; Enzinger, P. C.; Bang, Y. J.; Levitan, D.; Wang, J.; Rosales, M.; Dalal, R. P.; Yoon, H. H., Safety and Efficacy of Pembrolizumab Monotherapy in Patients With Previously Treated Advanced Gastric and Gastroesophageal Junction Cancer: Phase 2 Clinical KEYNOTE-059 Trial. *JAMA Oncol* **2018,** *4* (5), e180013.
8. Cohen, E. E. W.; Soulières, D.; Le Tourneau, C.; Dinis, J.; Licitra, L.; Ahn, M. J.; Soria, A.; Machiels, J. P.; Mach, N.; Mehra, R.; Burtness, B.; Zhang, P.; Cheng, J.; Swaby, R. F.; Harrington, K. J., Pembrolizumab versus methotrexate, docetaxel, or cetuximab for recurrent or metastatic head-and-neck squamous cell carcinoma (KEYNOTE-040): a randomised, open-label, phase 3 study. *Lancet* **2019,** *393* (10167), 156-167.
9. Bauml, J.; Seiwert, T. Y.; Pfister, D. G.; Worden, F.; Liu, S. V.; Gilbert, J.; Saba, N. F.; Weiss, J.; Wirth, L.; Sukari, A.; Kang, H.; Gibson, M. K.; Massarelli, E.; Powell, S.; Meister, A.; Shu, X.; Cheng, J. D.; Haddad, R., Pembrolizumab for Platinum- and

Cetuximab-Refractory Head and Neck Cancer: Results From a Single-Arm, Phase II Study. *J Clin Oncol* **2017,** *35* (14), 1542-1549.
10. Seiwert, T. Y.; Burtness, B.; Mehra, R.; Weiss, J.; Berger, R.; Eder, J. P.; Heath, K.; McClanahan, T.; Lunceford, J.; Gause, C.; Cheng, J. D.; Chow, L. Q., Safety and clinical activity of pembrolizumab for treatment of recurrent or metastatic squamous cell carcinoma of the head and neck (KEYNOTE-012): an open-label, multicentre, phase 1b trial. *Lancet Oncol* **2016,** *17* (7), 956-965.
11. Finn, R. S.; Ryoo, B. Y.; Merle, P.; Kudo, M.; Bouattour, M.; Lim, H. Y.; Breder, V.; Edeline, J.; Chao, Y.; Ogasawara, S.; Yau, T.; Garrido, M.; Chan, S. L.; Knox, J.; Daniele, B.; Ebbinghaus, S. W.; Chen, E.; Siegel, A. B.; Zhu, A. X.; Cheng, A. L., Pembrolizumab As Second-Line Therapy in Patients With Advanced Hepatocellular Carcinoma in KEYNOTE-240: A Randomized, Double-Blind, Phase III Trial. *J Clin Oncol* **2020,** *38* (3), 193-202.
12. Zhu, A. X.; Finn, R. S.; Edeline, J.; Cattan, S.; Ogasawara, S.; Palmer, D.; Verslype, C.; Zagonel, V.; Fartoux, L.; Vogel, A.; Sarker, D.; Verset, G.; Chan, S. L.; Knox, J.; Daniele, B.; Webber, A. L.; Ebbinghaus, S. W.; Ma, J.; Siegel, A. B.; Cheng, A. L.; Kudo, M., Pembrolizumab in patients with advanced hepatocellular carcinoma previously treated with sorafenib (KEYNOTE-224): a non-randomised, open-label phase 2 trial. *Lancet Oncol* **2018,** *19* (7), 940-952.
13. Schachter, J.; Ribas, A.; Long, G. V.; Arance, A.; Grob, J. J.; Mortier, L.; Daud, A.; Carlino, M. S.; McNeil, C.; Lotem, M.; Larkin, J.; Lorigan, P.; Neyns, B.; Blank, C.; Petrella, T. M.; Hamid, O.; Zhou, H.; Ebbinghaus, S.; Ibrahim, N.; Robert, C., Pembrolizumab versus ipilimumab for advanced melanoma: final overall survival results of a multicentre, randomised, open-label phase 3 study (KEYNOTE-006). *Lancet* **2017,** *390* (10105), 1853-1862.
14. Long, G. V.; Dummer, R.; Hamid, O.; Gajewski, T. F.; Caglevic, C.; Dalle, S.; Arance, A.; Carlino, M. S.; Grob, J. J.; Kim, T. M.; Demidov, L.; Robert, C.; Larkin, J.; Anderson, J. R.; Maleski, J.; Jones, M.; Diede, S. J.; Mitchell, T. C., Epacadostat plus pembrolizumab versus placebo plus pembrolizumab in patients with unresectable or metastatic melanoma (ECHO-301/KEYNOTE-252): a phase 3, randomised, double-blind study. *Lancet Oncol* **2019,** *20* (8), 1083-1097.
15. Ribas, A.; Puzanov, I.; Dummer, R.; Schadendorf, D.; Hamid, O.; Robert, C.; Hodi, F. S.; Schachter, J.; Pavlick, A. C.; Lewis, K. D.; Cranmer, L. D.; Blank, C. U.; O'Day, S. J.; Ascierto, P. A.; Salama, A. K.; Margolin, K. A.; Loquai, C.; Eigentler, T. K.; Gangadhar, T. C.; Carlino, M. S.; Agarwala, S. S.; Moschos, S. J.; Sosman, J. A.; Goldinger, S. M.; Shapira-Frommer, R.; Gonzalez, R.; Kirkwood, J. M.; Wolchok, J. D.; Eggermont, A.; Li, X. N.; Zhou, W.; Zernhelt, A. M.; Lis, J.; Ebbinghaus, S.; Kang, S. P.; Daud, A., Pembrolizumab versus investigator-choice chemotherapy for ipilimumab-refractory melanoma (KEYNOTE-002): a randomised, controlled, phase 2 trial. *Lancet Oncol* **2015,** *16* (8), 908-18.
16. Robert, C.; Ribas, A.; Schachter, J.; Arance, A.; Grob, J. J.; Mortier, L.; Daud, A.; Carlino, M. S.; McNeil, C. M.; Lotem, M.; Larkin, J. M. G.; Lorigan, P.; Neyns, B.; Blank, C. U.; Petrella, T. M.; Hamid, O.; Su, S. C.; Krepler, C.; Ibrahim, N.; Long, G. V., Pembrolizumab versus ipilimumab in advanced melanoma (KEYNOTE-006): post-hoc 5-year results from an open-label, multicentre, randomised, controlled, phase 3 study. *Lancet Oncol* **2019,** *20* (9), 1239-1251.
17. Robert, C.; Ribas, A.; Wolchok, J. D.; Hodi, F. S.; Hamid, O.; Kefford, R.; Weber, J. S.; Joshua, A. M.; Hwu, W. J.; Gangadhar, T. C.; Patnaik, A.; Dronca, R.; Zarour, H.; Joseph, R. W.; Boasberg, P.; Chmielowski, B.; Mateus, C.; Postow, M. A.; Gergich, K.; Elassaiss-Schaap, J.; Li, X. N.; Iannone, R.; Ebbinghaus, S. W.; Kang, S. P.;

Daud, A., Anti-programmed-death-receptor-1 treatment with pembrolizumab in ipilimumab-refractory advanced melanoma: a randomised dose-comparison cohort of a phase 1 trial. *Lancet* **2014,** *384* (9948), 1109-17.
18. Rossi, E.; Pagliara, M. M.; Orteschi, D.; Dosa, T.; Sammarco, M. G.; Caputo, C. G.; Petrone, G.; Rindi, G.; Zollino, M.; Blasi, M. A.; Cassano, A.; Bria, E.; Tortora, G.; Schinzari, G., Pembrolizumab as first-line treatment for metastatic uveal melanoma. *Cancer Immunol Immunother* **2019,** *68* (7), 1179-1185.
19. Popat, S.; Curioni-Fontecedro, A.; Dafni, U.; Shah, R.; O'Brien, M.; Pope, A.; Fisher, P.; Spicer, J.; Roy, A.; Gilligan, D.; Gautschi, O.; Nadal, E.; Janthur, W. D.; López Castro, R.; García Campelo, R.; Rusakiewicz, S.; Letovanec, I.; Polydoropoulou, V.; Roschitzki-Voser, H.; Ruepp, B.; Gasca-Ruchti, A.; Peters, S.; Stahel, R. A., A multicentre randomised phase III trial comparing pembrolizumab versus single-agent chemotherapy for advanced pre-treated malignant pleural mesothelioma: the European Thoracic Oncology Platform (ETOP 9-15) PROMISE-meso trial. *Ann Oncol* **2020,** *31* (12), 1734-1745.
20. Alley, E. W.; Lopez, J.; Santoro, A.; Morosky, A.; Saraf, S.; Piperdi, B.; van Brummelen, E., Clinical safety and activity of pembrolizumab in patients with malignant pleural mesothelioma (KEYNOTE-028): preliminary results from a non-randomised, open-label, phase 1b trial. *Lancet Oncol* **2017,** *18* (5), 623-630.
21. Yap, T. A.; Nakagawa, K.; Fujimoto, N.; Kuribayashi, K.; Guren, T. K.; Calabrò, L.; Shapira-Frommer, R.; Gao, B.; Kao, S.; Matos, I.; Planchard, D.; Chatterjee, A.; Jin, F.; Norwood, K.; Kindler, H. L., Efficacy and safety of pembrolizumab in patients with advanced mesothelioma in the open-label, single-arm, phase 2 KEYNOTE-158 study. *Lancet Respir Med* **2021,** *9* (6), 613-621.
22. McDermott, D. F.; Lee, J. L.; Ziobro, M.; Suarez, C.; Langiewicz, P.; Matveev, V. B.; Wiechno, P.; Gafanov, R. A.; Tomczak, P.; Pouliot, F.; Donskov, F.; Alekseev, B. Y.; Shin, S. J.; Bjarnason, G. A.; Castellano, D.; Silverman, R. K.; Perini, R. F.; Schloss, C.; Atkins, M. B., Open-Label, Single-Arm, Phase II Study of Pembrolizumab Monotherapy as First-Line Therapy in Patients With Advanced Non-Clear Cell Renal Cell Carcinoma. *J Clin Oncol* **2021,** *39* (9), 1029-1039.
23. Leighl, N. B.; Hellmann, M. D.; Hui, R.; Carcereny, E.; Felip, E.; Ahn, M. J.; Eder, J. P.; Balmanoukian, A. S.; Aggarwal, C.; Horn, L.; Patnaik, A.; Gubens, M.; Ramalingam, S. S.; Lubiniecki, G. M.; Zhang, J.; Piperdi, B.; Garon, E. B., Pembrolizumab in patients with advanced non-small-cell lung cancer (KEYNOTE-001): 3-year results from an open-label, phase 1 study. *Lancet Respir Med* **2019,** *7* (4), 347-357.
24. Theelen, W.; Chen, D.; Verma, V.; Hobbs, B. P.; Peulen, H. M. U.; Aerts, J.; Bahce, I.; Niemeijer, A. L. N.; Chang, J. Y.; de Groot, P. M.; Nguyen, Q. N.; Comeaux, N. I.; Simon, G. R.; Skoulidis, F.; Lin, S. H.; He, K.; Patel, R.; Heymach, J.; Baas, P.; Welsh, J. W., Pembrolizumab with or without radiotherapy for metastatic non-small-cell lung cancer: a pooled analysis of two randomised trials. *Lancet Respir Med* **2021,** *9* (5), 467-475.
25. Matulonis, U. A.; Shapira-Frommer, R.; Santin, A. D.; Lisyanskaya, A. S.; Pignata, S.; Vergote, I.; Raspagliesi, F.; Sonke, G. S.; Birrer, M.; Provencher, D. M.; Sehouli, J.; Colombo, N.; González-Martín, A.; Oaknin, A.; Ottevanger, P. B.; Rudaitis, V.; Katchar, K.; Wu, H.; Keefe, S.; Ruman, J.; Ledermann, J. A., Antitumor activity and safety of pembrolizumab in patients with advanced recurrent ovarian cancer: results from the phase II KEYNOTE-100 study. *Ann Oncol* **2019,** *30* (7), 1080-1087.
26. Strosberg, J.; Mizuno, N.; Doi, T.; Grande, E.; Delord, J. P.; Shapira-Frommer, R.; Bergsland, E.; Shah, M.; Fakih, M.; Takahashi, S.; Piha-Paul, S. A.; O'Neil, B.; Thomas, S.; Lolkema, M. P.; Chen, M.; Ibrahim, N.; Norwood, K.; Hadoux, J., Efficacy and Safety

of Pembrolizumab in Previously Treated Advanced Neuroendocrine Tumors: Results From the Phase II KEYNOTE-158 Study. *Clin Cancer Res* **2020,** *26* (9), 2124-2130.
27. Mehnert, J. M.; Bergsland, E.; O'Neil, B. H.; Santoro, A.; Schellens, J. H. M.; Cohen, R. B.; Doi, T.; Ott, P. A.; Pishvaian, M. J.; Puzanov, I.; Aung, K. L.; Hsu, C.; Le Tourneau, C.; Hollebecque, A.; Élez, E.; Tamura, K.; Gould, M.; Yang, P.; Stein, K.; Piha-Paul, S. A., Pembrolizumab for the treatment of programmed death-ligand 1-positive advanced carcinoid or pancreatic neuroendocrine tumors: Results from the KEYNOTE-028 study. *Cancer* **2020,** *126* (13), 3021-3030.
28. Antonarakis, E. S.; Piulats, J. M.; Gross-Goupil, M.; Goh, J.; Ojamaa, K.; Hoimes, C. J.; Vaishampayan, U.; Berger, R.; Sezer, A.; Alanko, T.; de Wit, R.; Li, C.; Omlin, A.; Procopio, G.; Fukasawa, S.; Tabata, K. I.; Park, S. H.; Feyerabend, S.; Drake, C. G.; Wu, H.; Qiu, P.; Kim, J.; Poehlein, C.; de Bono, J. S., Pembrolizumab for Treatment-Refractory Metastatic Castration-Resistant Prostate Cancer: Multicohort, Open-Label Phase II KEYNOTE-199 Study. *J Clin Oncol* **2020,** *38* (5), 395-405.
29. Raj, N.; Zheng, Y.; Kelly, V.; Katz, S. S.; Chou, J.; Do, R. K. G.; Capanu, M.; Zamarin, D.; Saltz, L. B.; Ariyan, C. E.; Untch, B. R.; O'Reilly, E. M.; Gopalan, A.; Berger, M. F.; Olino, K.; Segal, N. H.; Reidy-Lagunes, D. L., PD-1 Blockade in Advanced Adrenocortical Carcinoma. *J Clin Oncol* **2020,** *38* (1), 71-80.
30. Habra, M. A.; Stephen, B.; Campbell, M.; Hess, K.; Tapia, C.; Xu, M.; Rodon Ahnert, J.; Jimenez, C.; Lee, J. E.; Perrier, N. D.; Boraddus, R. R.; Pant, S.; Subbiah, V.; Hong, D. S.; Zarifa, A.; Fu, S.; Karp, D. D.; Meric-Bernstam, F.; Naing, A., Phase II clinical trial of pembrolizumab efficacy and safety in advanced adrenocortical carcinoma. *J Immunother Cancer* **2019,** *7* (1), 253.
31. Naing, A.; Meric-Bernstam, F.; Stephen, B.; Karp, D. D.; Hajjar, J.; Rodon Ahnert, J.; Piha-Paul, S. A.; Colen, R. R.; Jimenez, C.; Raghav, K. P.; Ferrarotto, R.; Tu, S. M.; Campbell, M.; Wang, L.; Sabir, S. H.; Tapia, C.; Bernatchez, C.; Frumovitz, M.; Tannir, N.; Ravi, V.; Khan, S.; Painter, J. M.; Abonofal, A.; Gong, J.; Alshawa, A.; McQuinn, L. M.; Xu, M.; Ahmed, S.; Subbiah, V.; Hong, D. S.; Pant, S.; Yap, T. A.; Tsimberidou, A. M.; Dumbrava, E. E. I.; Janku, F.; Fu, S.; Simon, R. M.; Hess, K. R.; Varadhachary, G. R.; Habra, M. A., Phase 2 study of pembrolizumab in patients with advanced rare cancers. *J Immunother Cancer* **2020,** *8* (1).
32. Tsimberidou, A. M.; Vo, H. H.; Subbiah, V.; Janku, F.; Piha-Paul, S.; Yilmaz, B.; Gong, J.; Naqvi, M. F.; Tu, S. M.; Campbell, M.; Meric-Bernstam, F.; Naing, A., Pembrolizumab in Patients with Advanced Metastatic Germ Cell Tumors. *Oncologist* **2021,** *26* (7), 558-e1098.
33. Tawbi, H. A.; Burgess, M.; Bolejack, V.; Van Tine, B. A.; Schuetze, S. M.; Hu, J.; D'Angelo, S.; Attia, S.; Riedel, R. F.; Priebat, D. A.; Movva, S.; Davis, L. E.; Okuno, S. H.; Reed, D. R.; Crowley, J.; Butterfield, L. H.; Salazar, R.; Rodriguez-Canales, J.; Lazar, A. J.; Wistuba, II; Baker, L. H.; Maki, R. G.; Reinke, D.; Patel, S., Pembrolizumab in advanced soft-tissue sarcoma and bone sarcoma (SARC028): a multicentre, two-cohort, single-arm, open-label, phase 2 trial. *Lancet Oncol* **2017,** *18* (11), 1493-1501.
34. Toulmonde, M.; Penel, N.; Adam, J.; Chevreau, C.; Blay, J. Y.; Le Cesne, A.; Bompas, E.; Piperno-Neumann, S.; Cousin, S.; Grellety, T.; Ryckewaert, T.; Bessede, A.; Ghiringhelli, F.; Pulido, M.; Italiano, A., Use of PD-1 Targeting, Macrophage Infiltration, and IDO Pathway Activation in Sarcomas: A Phase 2 Clinical Trial. *JAMA Oncol* **2018,** *4* (1), 93-97.
35. Cho, J.; Kim, H. S.; Ku, B. M.; Choi, Y. L.; Cristescu, R.; Han, J.; Sun, J. M.; Lee, S. H.; Ahn, J. S.; Park, K.; Ahn, M. J., Pembrolizumab for Patients With Refractory or Relapsed Thymic Epithelial Tumor: An Open-Label Phase II Trial. *J Clin Oncol* **2019,** *37* (24), 2162-2170.

36. Adams, S.; Schmid, P.; Rugo, H. S.; Winer, E. P.; Loirat, D.; Awada, A.; Cescon, D. W.; Iwata, H.; Campone, M.; Nanda, R.; Hui, R.; Curigliano, G.; Toppmeyer, D.; O'Shaughnessy, J.; Loi, S.; Paluch-Shimon, S.; Tan, A. R.; Card, D.; Zhao, J.; Karantza, V.; Cortés, J., Pembrolizumab monotherapy for previously treated metastatic triple-negative breast cancer: cohort A of the phase II KEYNOTE-086 study. *Ann Oncol* **2019,** *30* (3), 397-404.
37. Winer, E. P.; Lipatov, O.; Im, S. A.; Goncalves, A.; Muñoz-Couselo, E.; Lee, K. S.; Schmid, P.; Tamura, K.; Testa, L.; Witzel, I.; Ohtani, S.; Turner, N.; Zambelli, S.; Harbeck, N.; Andre, F.; Dent, R.; Zhou, X.; Karantza, V.; Mejia, J.; Cortes, J., Pembrolizumab versus investigator-choice chemotherapy for metastatic triple-negative breast cancer (KEYNOTE-119): a randomised, open-label, phase 3 trial. *Lancet Oncol* **2021,** *22* (4), 499-511.
38. Fradet, Y.; Bellmunt, J.; Vaughn, D. J.; Lee, J. L.; Fong, L.; Vogelzang, N. J.; Climent, M. A.; Petrylak, D. P.; Choueiri, T. K.; Necchi, A.; Gerritsen, W.; Gurney, H.; Quinn, D. I.; Culine, S.; Sternberg, C. N.; Nam, K.; Frenkl, T. L.; Perini, R. F.; de Wit, R.; Bajorin, D. F., Randomized phase III KEYNOTE-045 trial of pembrolizumab versus paclitaxel, docetaxel, or vinflunine in recurrent advanced urothelial cancer: results of >2 years of follow-up. *Ann Oncol* **2019,** *30* (6), 970-976.
39. Balar, A. V.; Castellano, D.; O'Donnell, P. H.; Grivas, P.; Vuky, J.; Powles, T.; Plimack, E. R.; Hahn, N. M.; de Wit, R.; Pang, L.; Savage, M. J.; Perini, R. F.; Keefe, S. M.; Bajorin, D.; Bellmunt, J., First-line pembrolizumab in cisplatin-ineligible patients with locally advanced and unresectable or metastatic urothelial cancer (KEYNOTE-052): a multicentre, single-arm, phase 2 study. *Lancet Oncol* **2017,** *18* (11), 1483-1492.
40. Galsky, M. D.; Mortazavi, A.; Milowsky, M. I.; George, S.; Gupta, S.; Fleming, M. T.; Dang, L. H.; Geynisman, D. M.; Walling, R.; Alter, R. S.; Kassar, M.; Wang, J.; Gupta, S.; Davis, N.; Picus, J.; Philips, G.; Quinn, D. I.; Haines, G. K., 3rd; Hahn, N. M.; Zhao, Q.; Yu, M.; Pal, S. K., Randomized Double-Blind Phase II Study of Maintenance Pembrolizumab Versus Placebo After First-Line Chemotherapy in Patients With Metastatic Urothelial Cancer. *J Clin Oncol* **2020,** *38* (16), 1797-1806.
41. Zhang, T.; Harrison, M. R.; O'Donnell, P. H.; Alva, A. S.; Hahn, N. M.; Appleman, L. J.; Cetnar, J.; Burke, J. M.; Fleming, M. T.; Milowsky, M. I.; Mortazavi, A.; Shore, N.; Sonpavde, G. P.; Schmidt, E. V.; Bitman, B.; Munugalavadla, V.; Izumi, R.; Patel, P.; Staats, J.; Chan, C.; Weinhold, K. J.; George, D. J., A randomized phase 2 trial of pembrolizumab versus pembrolizumab and acalabrutinib in patients with platinum-resistant metastatic urothelial cancer. *Cancer* **2020,** *126* (20), 4485-4497.
42. Plimack, E. R.; Bellmunt, J.; Gupta, S.; Berger, R.; Chow, L. Q.; Juco, J.; Lunceford, J.; Saraf, S.; Perini, R. F.; O'Donnell, P. H., Safety and activity of pembrolizumab in patients with locally advanced or metastatic urothelial cancer (KEYNOTE-012): a non-randomised, open-label, phase 1b study. *Lancet Oncol* **2017,** *18* (2), 212-220.
43. Powles, T.; Csőszi, T.; Özgüroğlu, M.; Matsubara, N.; Géczi, L.; Cheng, S. Y.; Fradet, Y.; Oudard, S.; Vulsteke, C.; Morales Barrera, R.; Fléchon, A.; Gunduz, S.; Loriot, Y.; Rodriguez-Vida, A.; Mamtani, R.; Yu, E. Y.; Nam, K.; Imai, K.; Homet Moreno, B.; Alva, A., Pembrolizumab alone or combined with chemotherapy versus chemotherapy as first-line therapy for advanced urothelial carcinoma (KEYNOTE-361): a randomised, open-label, phase 3 trial. *Lancet Oncol* **2021,** *22* (7), 931-945.
44. Piha-Paul, S. A.; Oh, D. Y.; Ueno, M.; Malka, D.; Chung, H. C.; Nagrial, A.; Kelley, R. K.; Ros, W.; Italiano, A.; Nakagawa, K.; Rugo, H. S.; de Braud, F.; Varga, A. I.; Hansen, A.; Wang, H.; Krishnan, S.; Norwood, K. G.; Doi, T., Efficacy and safety of

pembrolizumab for the treatment of advanced biliary cancer: Results from the KEYNOTE-158 and KEYNOTE-028 studies. *Int J Cancer* **2020,** *147* (8), 2190-2198.
